# Integrated Metabolomics and Network Pharmacology Revealed Hong-Hua-Xiao-Yao Tablet’s Effect of Mediating Hormone Synthesis in the Treatment of Mammary Gland Hyperplasia

**DOI:** 10.3389/fphar.2022.788019

**Published:** 2022-02-01

**Authors:** Ziqing Gao, Rui Mi, Zhaoxi Cheng, Xiaofeng Li, Huawu Zeng, Gaosong Wu, Jing Zhao, Weidong Zhang, Ji Ye

**Affiliations:** ^1^ Institute of Interdisciplinary Integrative Medicine Research, Shanghai University of Traditional Chinese Medicine, Shanghai, China; ^2^ School of Pharmacy, Second Military Medical University, Shanghai, China; ^3^ School of Pharmacy, Fudan University, Shanghai, China

**Keywords:** Hong-Hua-Xiao-Yao tablet, mammary gland hyperplasia, traditional medicine, metabolomics, network pharmacology, pharmacological effects

## Abstract

Hong-Hua-Xiao-Yao Tablet (HHXYT) is a traditional Chinese medicine (TCM) formula that has been approved for the treatment of mammary gland hyperplasia (MGH), but its mechanism of action is unclear. In this study, a strategy that integrated metabolomics and network pharmacology was applied to systemically reveal the mechanism of HHXYT in the treatment of MGH. Our pharmacodynamic study indicated that the proliferation of mammary gland was inhibited in rats, and serum-level disorder of estradiol and progesterone was reversed after HHXYT treatment. 54 compounds absorbed in rat plasma were identified after administration of HHXYT. The serum metabolome revealed 58 endogenous differential metabolites, of which 31% were steroid lipids metabolites, with steroid hormone biosynthesis being the most significant metabolic module. 7 targets, 6 herbs, and 17 ingredients were found to play key roles in HHXYT’s treatment of MGH. 3 of the 7 key targets (CYP11A1, HSD3B2, and CYP17A1) were directly involved in androgen synthesis, while 2 targets (AR and ESR1) were receptors for the direct action of androgens and estrogens. Molecular docking was utilized to confirm the bindings between the 5 targets and their corresponding compounds. In an *in vitro* test, HHXYT (50 µg/ml) and its ingredient formononetin (3.2, 6.3, and 12.5 µM) were found to significantly reduce the increase of testosterone level induced by dexamethasone (10 µM) in thecal cells. In summary, this study illustrated that the mechanism of HHXYT’s treatment of MGH was to regulate hormone disorder. HHXYT could reduce estrogen-stimulated hyperplasia by inhibiting the production of its precursor androgen.

## 1 Introduction

The most common breast disease, mammary gland hyperplasia (MGH), is a proliferative lesion of mammary tissue accompanying by the lumps and pain symptoms of the breast. It has overtly interfered with the patient’s daily life (You et al., 2017; Li et al., 2018). In the past few years, it has been increasingly reported that the incidence of breast cancer in MGH patients is remarkably higher than that in average person (Hartmann et al., 2005), since the pathogenesis of MGH is similar to that of breast cancer, both of which are closely related to endocrine and hormone level disorders (Coussens and Pollard, 2011; Arendt and Kuperwasser, 2015). Hormone drugs are currently applied to the therapy of MGH to regulate internal hormone levels. However, due to its obvious side effects, such as gastrointestinal and cardiovascular problems, the safety and effectiveness of this approach are not optimistic (Wibowo et al., 2016).

As a traditional Chinese medicine (TCM) formula on MGH treatment in the clinic for years, Hong-Hua-Xiao-Yao Tablet (HHXYT) was composed of nine herbal medicines, including *Carthamus tinctorius* L. (Honghua), *Angelica sinensis* (Oliv.) Diels (Danggui), *Paeonia lactiflora* Pall. (Baishao), *Gleditsia sinensis* Lam. (Zaojiaoci), *Atractylodes macrocephala* Koidz. (Baizhu), *Wolfiporia cocos* (F.A. Wolf) Ryvarden & Gilb (Fuling), *Mentha canadensis* L. (Bohe), *Bupleurum marginatum* Wall. ex DC. (Zhuye Chaihu), and *Glycyrrhiza uralensis* Fisch. ex DC. (Gancao). Chemical profiling of HHXYT has been previously elucidated by high-performance liquid chromatography combined with mass spectrometry technique, and 55 constituents were characterized. Moreover, in our earlier study, 14 components that were identified as potential HHXYT quality markers were quantified using high-performance liquid chromatography-tandem triple quadrupole mass spectrometry (HPLC-QQQ-MS) technique (Mi et al., 2020). In clinical trials, HHXYT was found to be effective in the treatment of perimenopausal syndrome, ovarian syndrome, menstrual disorders, as well as mammary gland hyperplasia (Wang, 2012; Pei et al., 2016; Chen et al., 2020). However, the mechanism of its therapy was still unelucidated.

Metabolomics could integrally focus on alterations of endogenous metabolites to monitor and analyze metabolic disturbances for elucidating therapeutic mechanisms (Nicholson and Wilson, 2003). Moreover, compared with other omics techniques, it is more in line with the change of disease phenotype (Newgard, 2017). Network pharmacology performs network analysis of complex biological systems by constructing network models (Hopkins, 2007; Li and Zhang, 2013). The combined analysis of metabolomics and network pharmacology can characterize the overall changes of biological systems, which is in accordance with the holistic therapeutic concept of TCM. This strategy has been increasingly applied in the study of TCM pharmacology and has contributed to elucidating the mechanism of multi-pathway and multi-target of TCM (Xu et al., 2018; Wu, Zhang and Li, 2019; Zhao et al., 2019).

**FIGURE 1:**
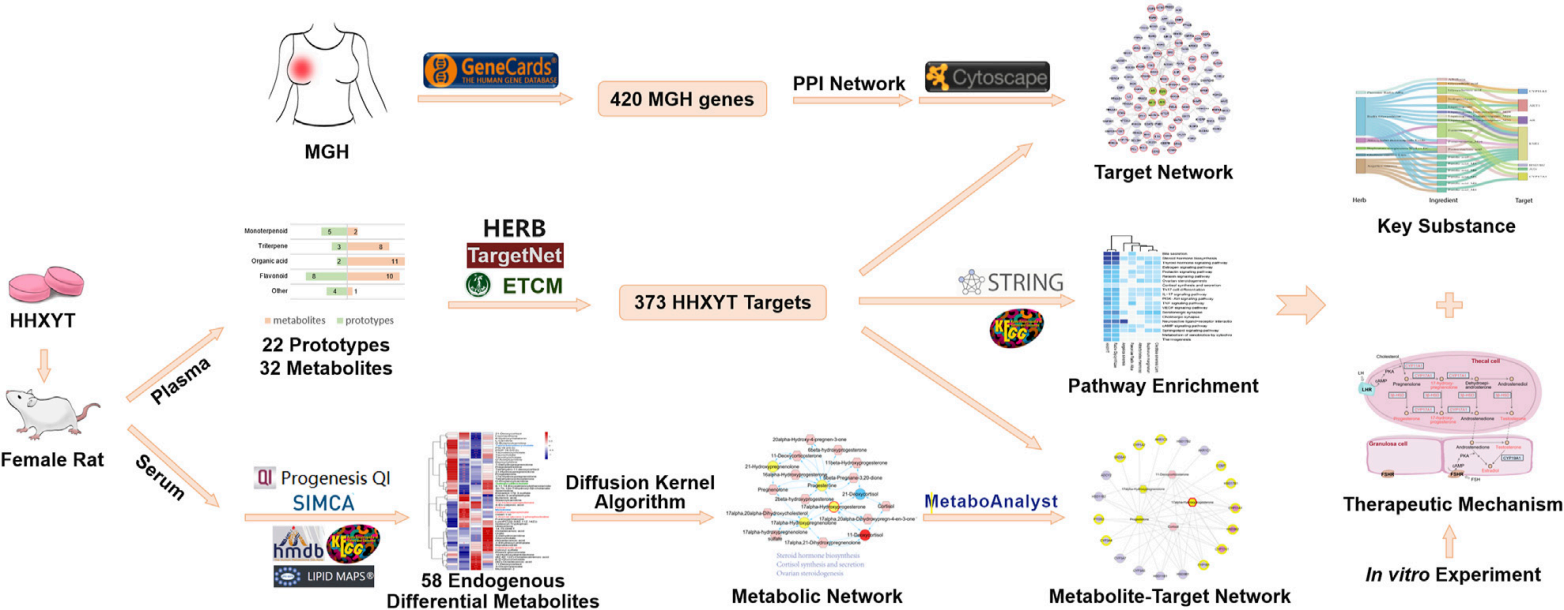
Integrated workflow for exploring the potential mechanisms of HHXYT against MGH.

In this study, the integrated metabolomics and network pharmacology strategy were used to reveal the mechanism of HHXYT in the treatment of MGH on a systemic level. Rat model of MGH was established and the metabolomics approach based on the HPLC-QTOF-MS/MS analysis was operated to search for serum endogenous differential metabolites. The ingredients and putative targets of HHXYT were then analyzed using the network pharmacology method. Next, an integrated analysis of differential metabolites and targets identified HHXYT’s key targets and substances against MGH. Ultimately, the cell-based assay on the synthesis of sex hormones was performed to validate HHXYT’s effects *in vitro* (Figure 1).

## 2 Materials and Methods

### 2.1 Chemicals and Reagents

Reagents of the LC-MS purity grade were acquired from Fisher Scientific (Fair Lawn, NJ, United Staes), including formic acid (purity >98%), acetonitrile, and methanol. Estradiol benzoate and progesterone injection were purchased from Zhejiang Xianju Pharmaceutical Co., Ltd. (Taizhou, China). ELISA kits of estradiol (E_2_), testosterone (T), and progesterone (P) were obtained from Boster Biological Technology Co., Ltd. (Shanghai, China). McCoy’s 5A medium was provided by Gibco^®^ (Grand Island, NY, United States). Follicle-stimulating hormone (FSH) and luteinizing hormone (LH) were acquired from Shanghai Linc-Bio Science Co. Ltd. (Shanghai, China). Dexamethasone (DEX) and formononetin (FT) were supplied by Chengdu Must Biotechnology Co., Ltd. (Chengdu, China). The purity of FT was above 98%. Cell Counting Kit-8 (CCK-8) was purchased from Shanghai Beyotime Biotechnology Co., Ltd. (Shanghai, China). HHXYT (batch number: 190106), the same batch named as “PZ-5” in our previous study (Mi et al., 2020), was supplied by Puzheng Pharmaceutical Co., Ltd. (Jiangxi, China). The contents of fourteen quality marker compounds of HHXYT were quantified by HPLC-QQQ-MS (Table 1). The chromatograms and chemical structure were shown in Supplementary Figure S1.

**TABLE 1:** The contents of fourteen quality marker components of HHXYT (batch number: 190106, mg/g, Mean ± SD, *n* = 3).

Number	Analytes	Structure class	Contents
1	Protocatechuic acid	Organic acid	0.296 ± 0.015
2	Paeoniflorin	Monoterpenoid	8.360 ± 0.589
3	Liquiritin	Flavonoid	0.693 ± 0.037
4	Senkyunolide I	Other	0.110 ± 0.007
5	Isoliquiritin	Flavonoid	0.301 ± 0.020
6	Liquiritigenin	Flavonoid	0.143 ± 0.004
7	Isoliquiritigenin	Flavonoid	0.085 ± 0.005
8	Formononetin	Flavonoid	0.028 ± 0.005
9	Glycyrrhizic acid	Triterpene	9.840 ± 0.312
10	Glycyrrhetinic acid 3-*O*-glucuronide	Triterpene	0.037 ± 0.008
11	Licoricone	Flavonoid	0.009 ± 0.001
12	Z-ligustilide	Other	5.013 ± 0.303
13	Licoisoflavone B	Flavonoid	0.022 ± 0.002
14	Glycyrrhetinic acid	Triterpene	0.015 ± 0.002

### 2.2 Animal Experiment and Sample Preparation

All sexually mature and non-pregnant female Sprague-Dawley rats were obtained from Shanghai Xipuer-Bikai Experimental Animal Co. Ltd., Shanghai, China (License No.: SCXK-Shanghai-2018-0006). They were bred in a stabilized environment for 1 week with room temperature 25 ± 2°C, relative humidity 55 ± 5%, 12 h each of light and dark, and free access to drinking water and feed. All procedures were approved by the Animal Care and Use Committee of Second Military Medical University and abided by the Guide for the Care and Use of Laboratory Animals.

#### 2.2.1 Prototypes of HHXYT and Related Metabolites in Plasma

The animal experiment used to study the chemicals absorbed in rat plasma was previously described (Mi et al., 2020). Briefly, the HHXYT sample was suspended in 0.5% carboxymethylcellulose sodium (CMC-Na) and administered orally to twenty-five female rats at five time-set points of 0.5, 1, 2, 4, and 6 h, at a dose of 8.82 g/kg (the optimal dose among 4 different doses in the amount and intensity of target compounds, Supplementary Figure S2), respectively (*n* = 5). Five other rats received oral administration of an equivalent volume of 0.5% CMC-Na. The blood samples were gathered from the hepatic portal vein, and all the samples were centrifuged at 3,500 rpm for 10 min at 4°C. A four-fold volume of methanol was used to extract the supernatants by the protein precipitation approach. After centrifuging at 13,000 rpm for 10 min at 4°C, the supernatants were blow-dried and reconstituted with 100 µL of 75% methanol for further analysis.

#### 2.2.2 Pharmacodynamics and Metabolomics Study

Twenty-four female Rats were haphazardly and equally divided into four groups: sham operation group (Sham), the mammary gland hyperplasia model group (MGH), low dose group of HHXYT (HHXYT-L), and high dose group of HHXYT (HHXYT-H). The MGH model was achieved by injecting intramuscularly with estradiol benzoate (0.5 mg/kg/d) for 25 days and then with progesterone (5 mg/kg/d) for 5 days (Wang et al., 2011). Meanwhile, the Sham group was injected with saline injection (0.5 mg/kg/d). After modeling, the HHXYT-L group and HHXYT-H group were administrated HHXYT orally with suspending in 0.5% CMC-Na aqueous solution, at a dosage of 0.5 g/kg/d (clinical equivalent dose) and 4.5 g/kg/d for 3 weeks, respectively. The Sham group and MGH group were given oral administration of 0.5% CMC-Na.

Rats were injected intraperitoneally with 10% chloral hydrate for anesthesia after 3 weeks of administration, and the diameter of the second pair of nipples was measured. The blood samples were obtained from the abdominal aorta, centrifuged at 3,500 rpm at 4°C for 15 min. The serum was separated from the upper layer and stored at −80°C. The left mammary gland of the second pair of breasts was collected and fixed in tissue fixative for 24 h, then sectioned in paraffin and stained with Hematoxylin-Eosin (HE) for optical microscopy. The contents of estradiol (E_2_) and progesterone (P) in serum were determined by ELISA kits.

300 µL of methanol was put in 100 µL of serum, which was eddied for 1 min to ensure complete mingling before centrifugation at 14,000 rpm for 15 min at 4°C. The supernatant was aspirated and stored overnight at −80°C, followed by centrifugation under the same conditions for 15 min to remove proteins once again. The final supernatant was pipetted into the inner tube of vials and allowed to stand for LC-MC analysis. The quality control samples (QC) were prepared by pooling 20 µL of 6 random samples (containing all groups) from all samples.

### 2.3 Instrument Parameters

#### 2.3.1 UPLC-QTOF-MS/MS for Identifying Prototypes of HHXYT and Related Metabolites in Plasma

The compounds absorbed in rat plasma were detected using a Waters Acquity UPLC I-class system (Waters Corp., Milford, MA, United States) and Q-tof Synapt G2-Si mass spectrometry system (Waters, Manchester, United Kingdom). A Waters Acquity UPLC BEH reverse-phase C18 analytical column (1.7 µm, 3.0 × 100 mm, Waters) was used to separate the compounds. The column temperature was set at 35°C and the injection volume was 5 µL. The mobile phase was made up of 0.1% formic acid in water (*v/v*) (A) and acetonitrile (B). At a flow rate of 0.4 ml/min, the elution gradient was processed in 37 min. The base peak chromatograms of both positive and negative ion modes were acquired under multiple detection modes of MS^E^ and Fast DDA. The parameters were set following the previous report (Mi et al., 2020).

#### 2.3.2 HPLC-QTOF-MS/MS for Metabolomics Analysis

LC system was considered to apply with a Shimadzu HPLC system (Nexera XR LC-20AD, Japan) and use an ACQUITY UPLC^®^ HSS T3 (2.1 × 100 mm, 1.8 µm, Waters, Milford, MA, United States) as the chromatographic column. Mass spectrometric detection was carried out on a SCIEX Triple TOF 5600^+^, using the electrospray ion (ESI) source and information-dependent acquisition (IDA) mode. The column temperature was set at 40°C and the injection volume was 2 µL. The mobile phase was made up of mass spectrometric grade 0.1% formic acid in water (*v/v*) (A) and acetonitrile (B). The calibration deliver system (CDS) was applied to calibrate the mass spectrometric acquisitions automatically. Additional parameter settings are provided in the Supplementary Materials.

### 2.4 Data Processing for Metabolomics Results

The raw data files were imported into the Progenesis QI 2.0 software (Waters, Milford, MA, United States) for data preprocessing, including alignment, peak picking, and compound identification. StatTarget was utilized to remove system errors as well as signal drift (Luan et al., 2018). The data was then imported into SICMA 14.1 (Umetric, Umeå, Sweden) for multivariable statistical analysis. Variates with *p*-values < 0.05, fold change (FC) ≥1.2 or FC ≤ 0.8, and VIP >1 (variable importance in the projection), were considered as endogenous differential metabolites, and further identified with online databases of HMDB (Wishart et al., 2018), LIPID MAPS (Liebisch et al., 2020) and KEGG (Kanehisa et al., 2017).

### 2.5 Identification and Analysis of Metabolic Modules

We applied the diffusion kernel algorithm to detect metabolites that locate at the proximity of the endogenous differential metabolites in the genome-scale metabolic network of rats (Luan et al., 2018). The diffusion parameter was set as 0.1. All endogenous differential metabolites of the MGH, HHXYT-L, and HHXYT-H groups were input as seed nodes to perform the diffusion kernel algorithm. The nodes with high diffusion scores were taken as compounds close to the endogenous differential metabolites in the genome-scale metabolic network. Then, the metabolic network affected by mammary glands hyperplasia and HHXYT treatment was constructed by extracting these nodes and links between them from the background network.

### 2.6 Putative Targets for HHXYT’s Plasma Absorbed Compounds

For prototypes and their metabolites in the plasma of rats after intragastric administration of HHXYT, we collected or predicted their putative targets from 3 databases and web servers: ETCM (Encyclopaedia of Traditional Chinese Medicine) (Xu et al., 2019), HERB (High-throughput Experiment- and Reference-guided dataBase of traditional Chinese medicine) (Fang et al., 2021), and TargetNet (Yao et al., 2016). Target information in HERB was collected by manual reference mining and from multiple TCM databases, including SymMap (Wu, Zhang, Yang et al., 2019), TCMID (Xue et al., 2013), TCMSP (Ru et al., 2014), and TCM-ID (Chen et al., 2006). The threshold of confidence score was set as 0.9 to filter the compound-protein interactions predicted by TargetNet.

### 2.7 Collection of MGH Associated Genes and Functional Enrichment Analysis

Disease genes of MGH were collected from the GeneCards database (Stelzer et al., 2016). Inputting the keyword “mammary gland hyperplasia” generated 4,251 results with relevance scores in the area [0.53, 248.35]. We set the threshold of relevance score as 20 to filter the results and obtained 420 top-ranked genes with high relevance to MGH, taking about 10% of the results. These 420 genes were considered as MGH associated genes for further analysis.

We used the STRING platform to conduct functional enrichment analysis for gene groups under study (Szklarczyk et al., 2019).

### 2.8 Network Construction, Analysis, and Visualization

The genomic scale protein-protein interaction (PPI) network of human beings was downloaded from the HINT (High-quality INTeractomes) database (Das and Yu, 2012), whose PPI data was integrated from 8 databases (BioGRID, MINT, iRefWeb, DIP, IntAct, HPRD, MIPS, and the PDB) and filtered both systematically and manually to keep high-confidence and -quality interactions. It was used as a background network for the construction of HHXYT’s target network. We used Cytoscape version 3.6.0 to construct, analysis and visualize networks (Shannon et al., 2003). The Cytohubba app of Cytoscape was applied to compute topological measures of nodes (Chin et al., 2014).

Metabolite-gene network was constructed by the Network Analysis module of MetaboAnalyst platform version 5.0 (Chong et al., 2019).

### 2.9 Molecular Docking

The SDF file for a ligand’s structure was downloaded from the PubChem database (Kim et al., 2021). The PDB file for a protein receptor’s crystal structure was obtained from RCSB Protein Data Bank (PDB) database (Burley et al., 2021). For proteins without known crystal structures, the predicted crystal structures were downloaded from AlphaFold Protein Structure Database (Jumper et al., 2021). The preprocessing and preparing of ligands and receptors were conducted by AutoDock Tools (Tanchuk et al., 2016) following the tutorial and manual (http://vina.scripps.edu/manual.html). Each ligand was docked with corresponding receptors by AutoDock Vina (Trott and Olson, 2010), and a compound-target pair with the docking score of less than −5 kcal/mol was considered as a binding pair. PLIP platform was used to analyze the binding sites of the binding compound-target pair (Adasme et al., 2021). PyMol was applied for visualizing the results of AutoDock Vina and PLIP (Alexander et al., 2011).

### 2.10 Cell-Based Assay Evaluation of HHXYT *In Vitro*

Thecal cells were extracted and purified from the ovaries of female rats at 3–4 weeks old (Li and Hearn, 2000). 5 × 10^3^ thecal cells per well were seeded into 96-well plates and set as the control group and experimental groups. The control group was McCoy’s 5A medium containing thecal cell. The experimental groups were based on the control group supplemented with different concentrations of luteinizing hormone (LH), follicle-stimulating hormone (FSH), HHXYT, and its ingredient formononetin (FT). After 36 h of adherent culture at 37°C and 5% CO_2_, these drugs were supplemented according to different groups and cultured for another 24 h. Then 10 µL of CCK-8 reagent was added to each well in the dark, and the absorbance was measured at a wavelength of 450 nm 2 h later to calculate the cell survival rate (Chen et al., 2021).

4 × 10^4^ Cells were added to each well of 48-well plates containing McCoy’s 5A medium, which consisted of 10% FBS and 1% penicillin-streptomycin. After culturing at 37°C and 5% CO_2_ for 36 h, different concentrations of LH, dexamethasone (DEX), HHXYT, and FT were added into different groups of wells and cultured for another 24 h (Ong et al., 2019). ELISA kits were used to detect the levels of estradiol (E_2_) or testosterone (T) in cell media.

## 3 Results

### 3.1 Pharmacodynamic Results of HHXYT on MGH Model

**FIGURE 2:**
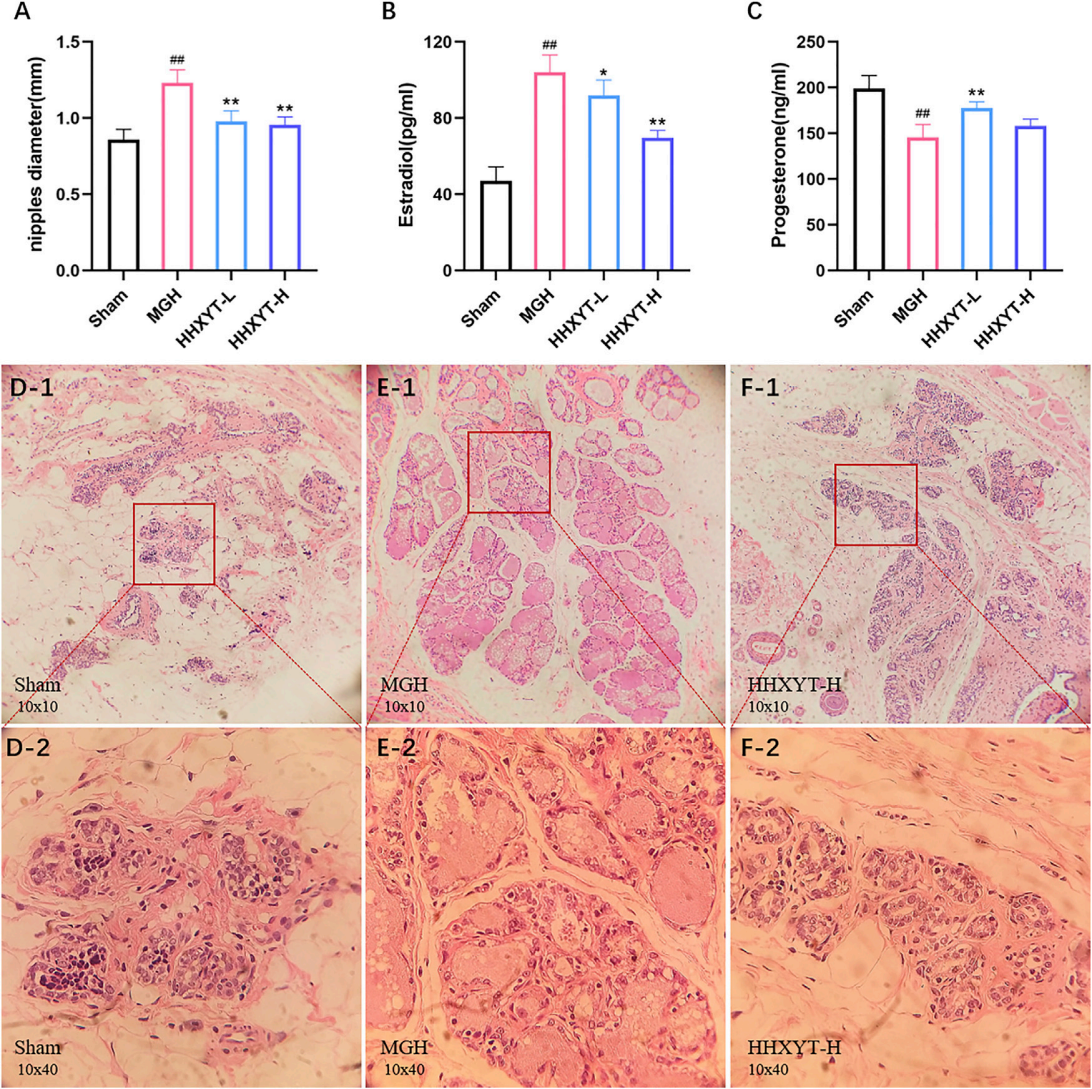
The effect of HHXYT on pharmacodynamic results of the MGH model. **(A)** The diameter of nipples. The serum levels of sex hormone estradiol **(B)** and progesterone **(C)**. *n* = 6 per group, ##*p* < 0.01, compared to Sham; *p < 0.05, **p < 0.01, compared to MGH. HE staining results for mammary gland sections in Sham **(D)**, MGH **(E)**, HHXYT-H **(F)** group (10 × 10: D-1, E-1, and F-1; 10 × 40: D-2, E-2, and F-2).

To evaluate the therapeutic effect of HHXYT on MGH, we detected variations of nipple diameter and serum sex hormone level. After establishing the MGH model, the nipple diameter grew significantly (*p* < 0.01, MGH vs. Sham) from 0.858 ± 0.068 mm to 1.230 ± 0.086 mm, compared to the Sham group. Different doses of HHXYT, on the other hand, could significantly (*p* < 0.01, HHXYT vs. MGH) reduce nipple enlargement (Figure 2A). Meanwhile, in the MGH group, the estradiol level in serum increased prominently (*p* < 0.01, MGH vs. Sham) while the progesterone level decreased notably (*p* < 0.01, MGH vs. Sham). After treatment with different doses of HHXYT, the serum level of estradiol and progesterone was reversed, respectively (Figures 2B,C).

According to Hematoxylin-Eosin (HE) staining, mammary tissue in the Sham group had no aberrant proliferation, a tiny number of the acinus, nearly no dilatation, and no secretion of mammary ducts and lumens (Figure 2D). The mammary tissue in the MGH group showed distinct proliferation; the volume of mammary lobules was increased; the number of glandular bubbles was also increased and became close to fusion; the acinus and lumens were revealed dilatation and excessive secretions (Figure 2E). However, after HHXYT treatment, mammary gland hyperplasia was prominently alleviated due to a considerable reduction in the account of the volume of mammary lobules, the number of glandular vesicles, and the secretion of lumens (Figure 2F).

### 3.2 Characterization of Prototypes and Metabolites in HHXYT, and Functional Enrichment Analysis of Putative Targets

**FIGURE 3:**
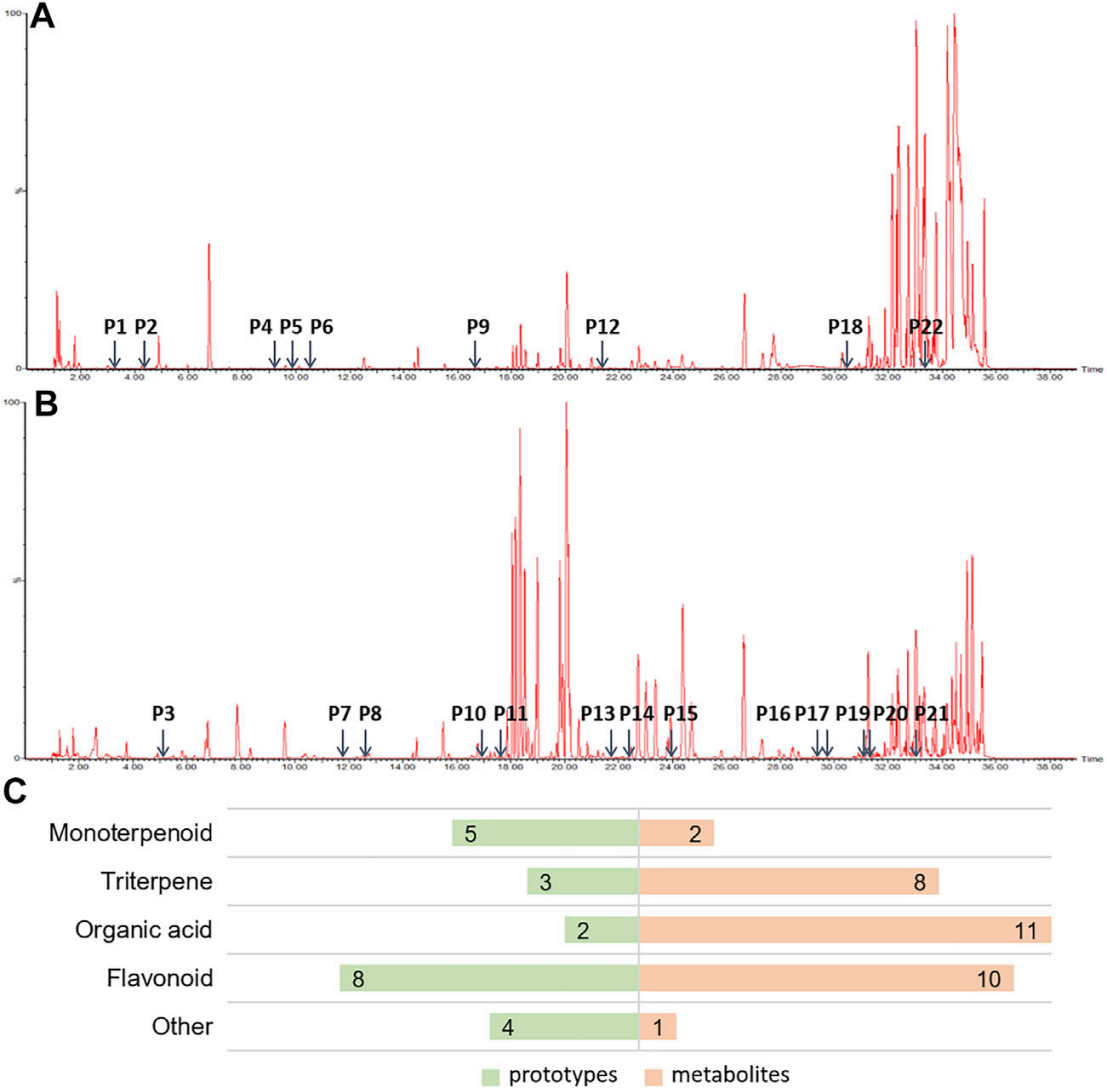
The prototypes and their metabolites in the plasma of rats after intragastric administration of HHXYT. Total ion chromatograms of rat plasma samples after intragastric administration of HHXYT and the distribution of 22 prototypes, positive mode **(A)** and negative mode **(B)**. **(C)** Categories of prototypes and metabolites.

It is widely accepted that prototypes and their metabolites in blood after oral TCM intake have a therapeutic effect (Ye et al., 2019). In order to determine the active components of HHXYT, we comprehensively analyzed the prototypes and their metabolites of HHXYT in rat plasma. A total of 54 components were identified, comprising 22 prototypes (Figures 3A,B; Supplementary Table S1) and 32 metabolites (Supplementary Table S2). After HHXYT treatment, three prototype components, named desbenzoylpaeoniflorin, 1-*O*-*β*-d–glucopyranosylpaeonisuffrone, and bohenoside A, as well as 32 metabolites, were identified in rat plasma for the first time. These 32 metabolites include 2 monoterpenoids, 8 triterpenes, 11 organic acids, 10 flavonoids, and 1 other metabolite (Figure 3C; Supplementary Table S4-S7).

Based on 54 active ingredients identified in the rat plasma, we predicted their potential target through network pharmacology (hydroxysafflower yellow A was also added to the analysis because it was reported to be an active ingredient of *Carthami Flos* (Honghua) (Li et al., 2021)). Using 3 databases and web servers (ETCM, HERB, and TargetNet), 373 distinct protein targets were predicted as HHXYT’s putative targets regulated by the 55 active ingredients (Supplementary Table S3).

The STRING platform was utilized to analyze the 373 targets for functional enrichment. Setting FDR<10^−4^ as the threshold, we identified 93 statistically enriched KEGG pathways. The top enriched pathways were presented in Table 2, and these pathways were mainly involved in the biological processes of the endocrine, immune, and nervous system.

**TABLE 2:** Enriched KEGG pathways of HHXYT’s targets. The pathways with bold type are associated with metabolic modules affected by HHXYT.

Pathway class	Pathway name	HHXYT targets	Total genes	FDR
Endocrine	**Bile secretion**	30	71	1.08E-25
	**Steroid hormone biosynthesis**	28	58	1.85E-25
	Thyroid hormone signaling pathway	29	115	2.59E-20
	Estrogen signaling pathway	24	133	2.01E-14
	Prolactin signaling pathway	19	69	2.42E-14
	Relaxin signaling pathway	22	130	6.79E-13
	**Ovarian steroidogenesis**	15	49	3.65E-12
	**Cortisol synthesis and secretion**	8	63	8.24E-05
Immune	Th17 cell differentiation	21	102	1.09E-13
	IL-17 signaling pathway	20	92	1.74E-13
	PI3K-Akt signaling pathway	37	348	3.66E-15
	TNF signaling pathway	23	108	4.18E-15
	VEGF signaling pathway	16	59	3.10E-12
Nervous system	Serotonergic synapse	24	112	1.06E-15
	Cholinergic synapse	20	111	2.97E-12
	Neuroactive ligand-receptor interaction	36	272	2.74E-17
	cAMP signaling pathway	30	195	4.60E-16
	Sphingolipid signaling pathway	23	116	1.39E-14
Other	Metabolism of xenobiotics by cytochrome P450	18	70	2.81E-13
	Thermogenesis	30	228	1.37E-14

**FIGURE 4:**
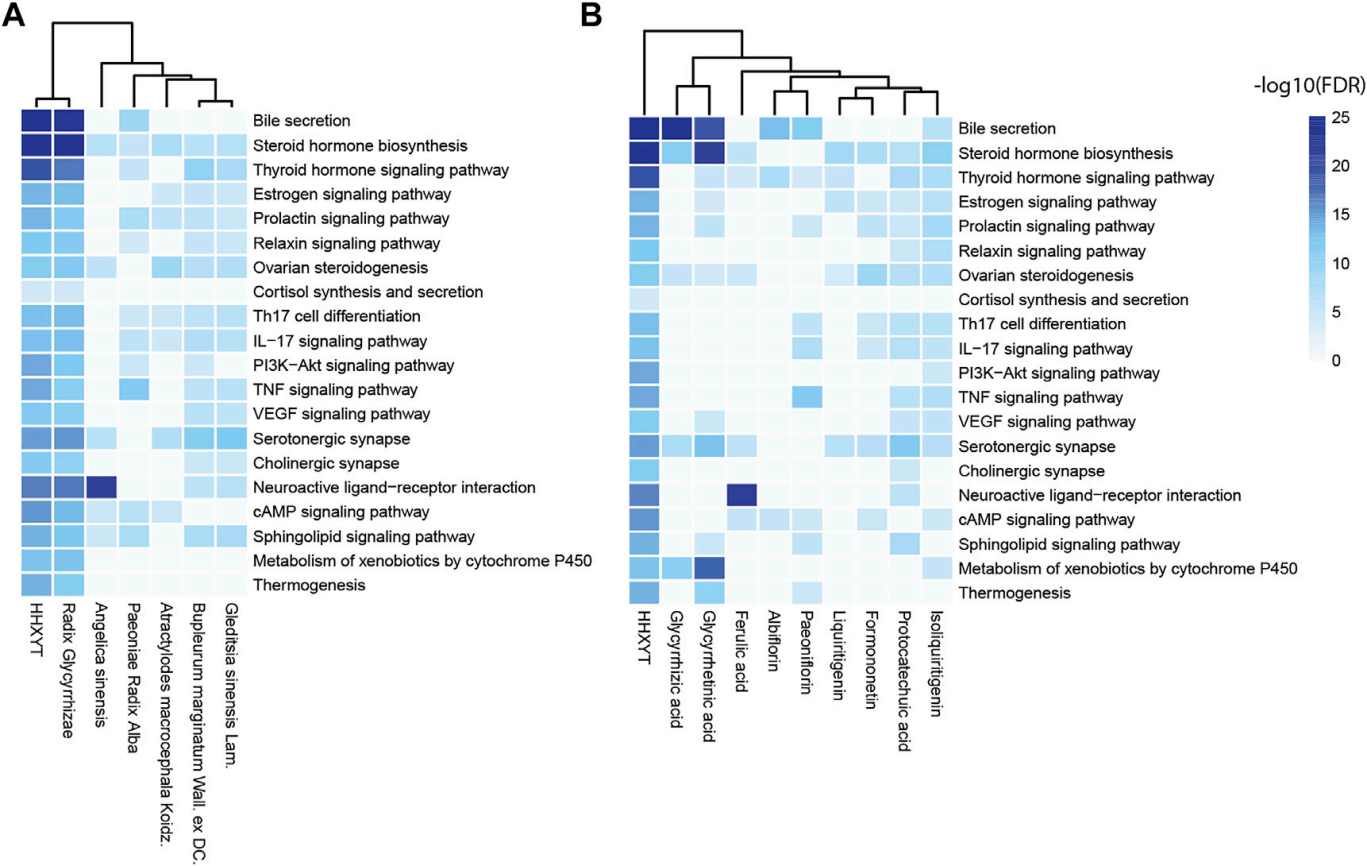
KEGG pathway enrichment analysis for the targets of herbs and prototypes of HHXYT. Targets of 6 herbs **(A)** and 9 prototypes **(B)** from HHXYT were enriched in at least 3 of the top 20 KEGG pathways regulated by HHXYT, respectively.

Then, based on the KEGG pathways in Table 2, we performed enrichment analysis for targets of HHXYT’s herbs and prototypes. When conducting enrichment analysis, we combined targets of metabolites derived from its prototype with targets of the prototype. The threshold for statistical significance was set as FDR<10^−4^. As shown in Figure 4, targets of 6 herbs and 9 prototypes from HHXYT were enriched in at least 3 of the 20 KEGG pathways in Table 2, respectively. These results suggested that the 6 herbs and 9 prototypes could play key roles in HHXYT’s treatment of MGH.

### 3.3 HHXYT Alters the Serum Metabolome

**FIGURE 5:**
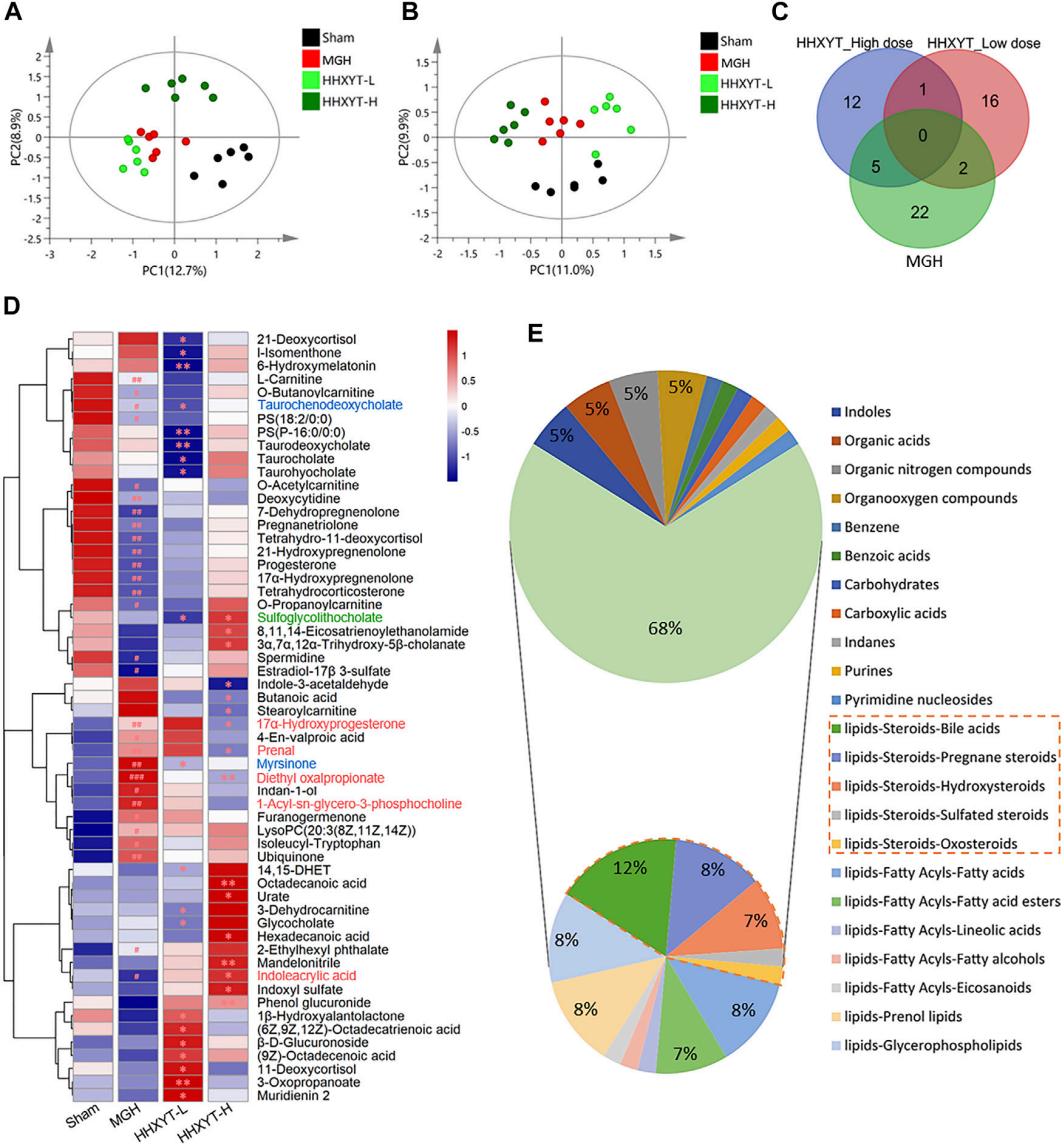
Endogenous differential metabolites were identified from the metabolomics experiment. PLS-DA score plots of Sham, MGH HHXYT-L, and HHXYT-H, in positive mode **(A)** and negative mode **(B)**. **(C)** The intersection of endogenous differential metabolites in the 3 groups. **(D)** Heatmap of 58 endogenous differential metabolites. *n* = 6, #*p* < 0.05, ##*p* < 0.01, compared to Sham; *p < 0.05, **p < 0.01, compared to MGH. The red compound name means the common differential metabolites between HHXYT-H and MGH; Blue: the common differential metabolites of HHXYT-L/MGH; Green: the common differential metabolites of HHXYT-H/HHXYT-L. **(E)** Categories of endogenous differential metabolites. 31% of steroids were marked with the orange border.

On the basis of research on prototypes and their metabolites of HHXYT, the metabolomics analysis was used to further identify the serum endogenous differential metabolites of rats intervened by HHXYT. The PLS-DA model was applied to characterize the metabolic disturbances. As shown in Figures 5A,B, the serum samples of Sham, MGH, HHXYT-L, and HHXYT-H groups were distinctly separated in both positive and negative modes. Based on the criteria of *p*-values < 0.05, FC ≥ 1.2 or FC ≤ 0.8, and VIP >1, 58 endogenous differential metabolites in serum were screened and identified, including 29 metabolites in the Sham/MGH group, 19 metabolites in the MGH/HHXYT-L group, and 18 metabolites in the MGH/HHXYT-H group (Figure 5C). There were 5 same endogenous differential metabolites between HHXYT-H and MGH (Figure 5D, red compound names), as well as 2 overlaps between HHXYT-L and MGH (blue compound names). Furthermore, the regulatory trends of HHXYT-H on the 5 same differential metabolites were all opposite to that of MGH. Meanwhile, only one differential metabolite had been oppositely regulated between HHXYT-L and MGH groups. These results indicated that the HHXYT-H group had more pronounced alterations in metabolites endogenous on treating MGH. The majority of these endogenous differential metabolites are lipid metabolites, accounting for 68%, including 31% steroids lipid (12% bile acids, 8% pregnane steroids, 7% hydroxysteroids, etc.), 8% fatty acids, 7% fatty acid esters, 8% prenol lipids, 8% glycerophospholipids and so on (Figure 5E). The above results suggested that HHXYT could prominently regulate lipid metabolism, especially hormone disorder in rats with hyperplasia of mammary glands.

### 3.4 Metabolic Modules Affected by the Treatment of HHXYT

**FIGURE 6:**
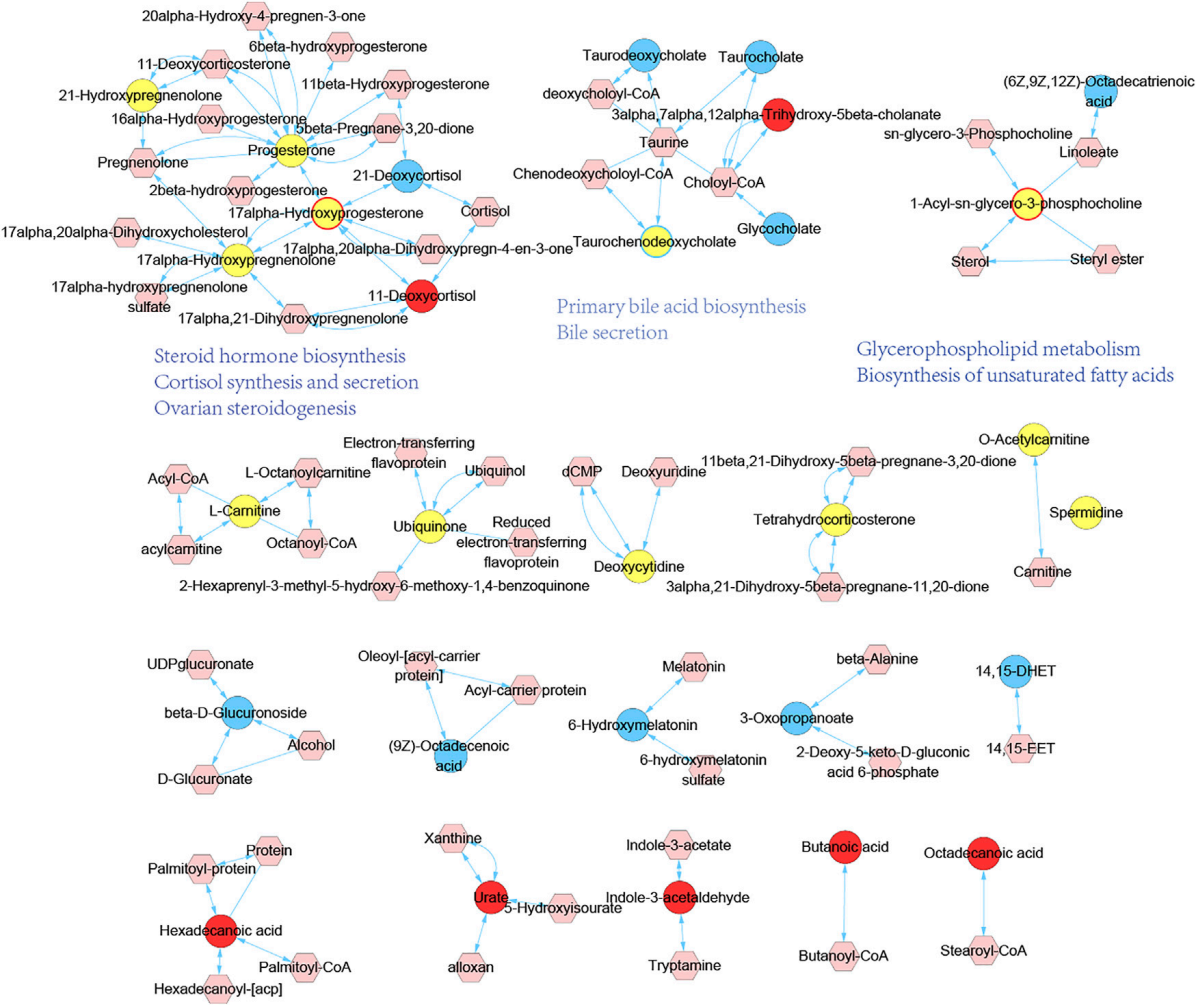
Metabolic modules affected by HHXYT treatment and their functions. Circle nodes are endogenous differential metabolites identified in metabolomics, and hexagonal nodes are metabolites close to the endogenous differential metabolites in the genomic metabolic network which were identified by the diffusion kernel algorithm. Yellow circles are endogenous differential metabolites in MGH, while circles with blue and red colors or borders were in HHXYT-L and HHXYT-H, respectively.

To reveal the underlying significance of metabolites variation *in vivo,* we proceeded with metabolic module establishment and pathway analysis of endogenous differential metabolites. Inputting all the endogenous differential metabolites as seed nodes, we ran the diffusion kernel algorithm to score all nodes in the genome-scale metabolic network of rats. The metabolic network influenced by MGH and HHXYT therapy was then constructed by extracting the nodes with diffusion scores at least 0.05 and the links between them (Figure 6). This network consists of 19 isolated modules, in which only the first three large modules include endogenous differential metabolites affected by both MGH and HHXYT treatment, suggesting that they could be key metabolic modules by which HHXYT exerts its therapeutic effect on MGH.

We searched the KEGG database (Kanehisa et al., 2017) to annotate the first 3 modules in Figure 6. It was found that metabolites in the first module were mainly involved in pathways of steroid hormone biosynthesis, cortisol synthesis and secretion, and ovarian steroidogenesis; the compounds in the second module participated in primary bile acid biosynthesis and bile secretion; the third module was associated with glycerophospholipid metabolism and biosynthesis of unsaturated fatty acids. Notably, all the 3 pathways associated with the first metabolic module and 1 pathway of the second metabolic module, were also found in the pathways listed in Table 2, which were enriched by 373 HHXYT’s predicted targets. It demonstrated that the pathways involved by HHXYT’s putative targets discovered by network pharmacology were consistent with the analyzed regulation of metabolic disturbances in rat serum by metabolomics.

### 3.5 Network Pharmacological Analysis for HHXYT’s Putative Targets and Metabolic Modules

**FIGURE 7:**
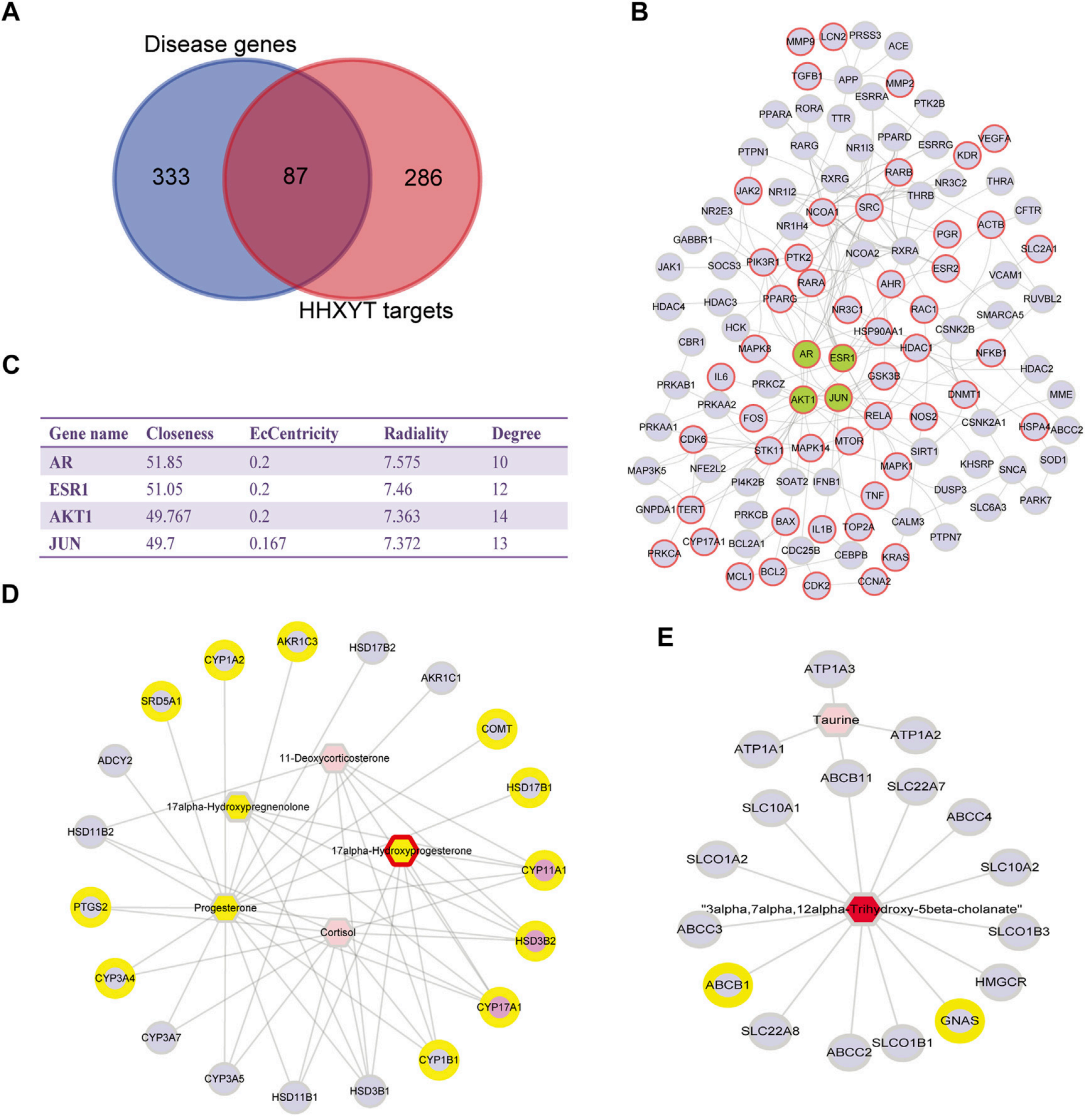
Network pharmacological analysis for HHXYT’s putative targets and integrated analysis of metabolic modules. **(A)** The intersection between HHXYT’s putative targets and MGH disease genes from GeneCards. **(B)** HHXYT’s putative target network, where the nodes with red borders are MGH disease genes and the green nodes are central nodes with the highest global centrality. **(C)** Global (Closeness, EcCentricity, Radiality) and local centrality measures (Degree) of the four central nodes in the target network. Metabolite-gene networks for the first **(D)** and second metabolic module **(E)** affected by HHXYT, respectively. Hexagons were endogenous differential metabolites identified from the metabolomics analysis and circles were HHXYT’s target and genes. Yellow represented in MGH disease conditions, while red represented in HHXYT-H. Pink circles were the hub genes of the network.

The first step was to conduct a network analysis of HHXYT’s potential targets. We calculated the intersections of HHXYT’s 373 target genes with the 420 diseases genes for MGH (Supplementary Table S4; Figure 7A). It could be seen that one-fourth of HHXYT’s targets were disease genes. Then we constructed the protein-protein interaction (PPI) network between the targets based on the human genomic-scale PPI network from the HINT database. As shown in Figure 7B, 114 of the 373 targets interacted with each other to form HHXYT’s target network, with 53 of them being disease genes associated with MGH.

The centrality measures for nodes in the target network were computed using the Cytohubba App of the Cytoscape software. The centrality of nodes was then ranked applying 3 global parameters based on shortest paths (Closeness, EcCentricity, and Radiality). As shown in Figure 7C, the 4 nodes with the highest global centrality were AR, ESR1, AKT1, and JUN, suggesting that they were central nodes whose impacts were easy to reach out into the whole target network.

The Network Analysis module of the MetaboAnalyst platform version 5.0 was then utilized to construct metabolite-gene networks corresponding to the metabolic modules affected by HHXYT. We input the endogenous differential metabolites in the first and second modules of Figure 6, together with HHXYT’s targets enriched in the corresponding pathways of the modules, respectively. Then we got the metabolite-gene interaction modules for the first and second metabolite module affected by HHXYT (Figures 7D,E). As shown in Figure 7D, the first module included 3 hub genes, i.e., CYP11A1, HSD3B2, and CYP17A1, which linked to all the metabolites in the network, implying that they performed a key role in HHXYT’s treatment to MGH.

**FIGURE 8:**
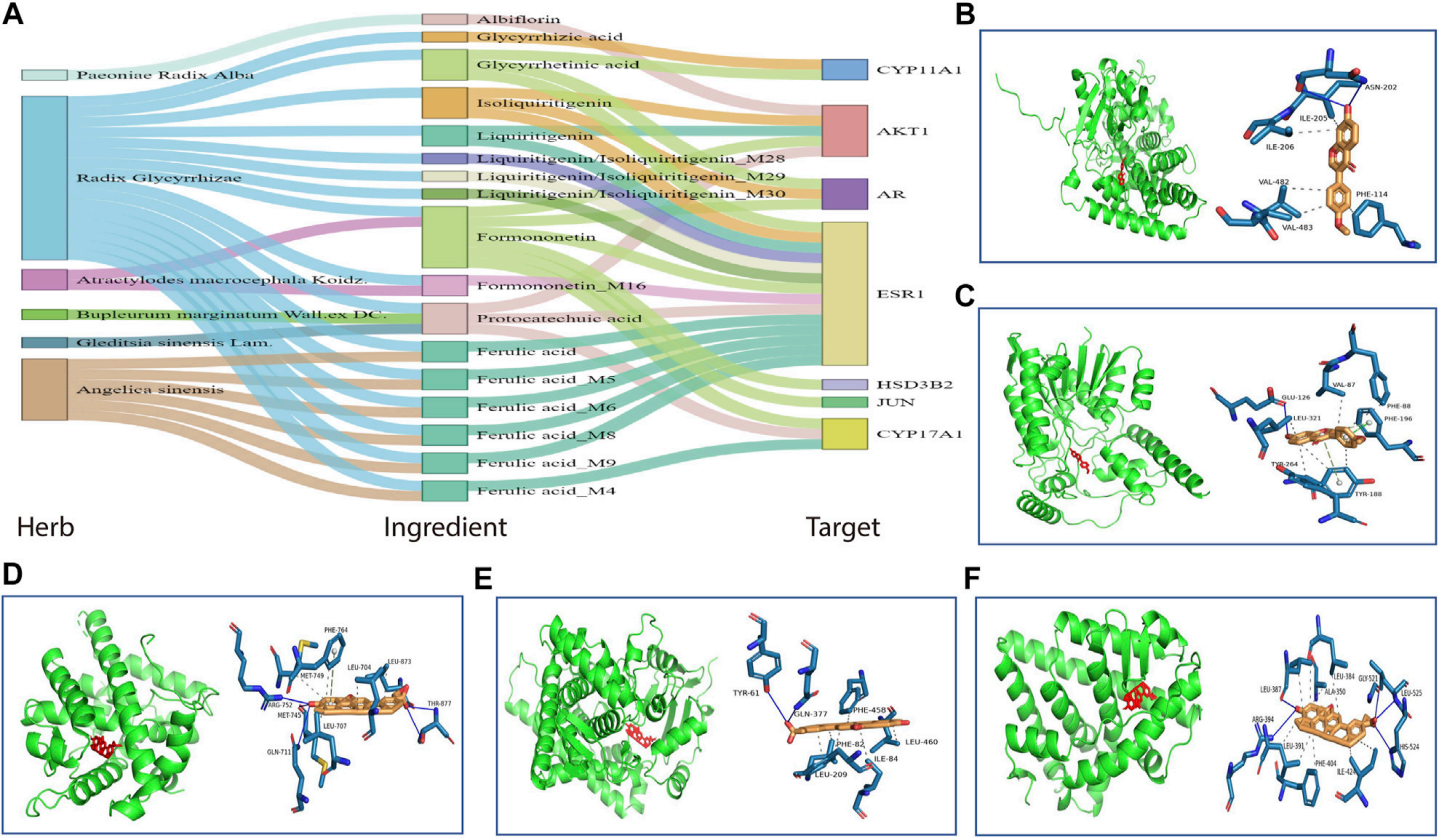
The key substances for HHXYT to exert therapeutic effects on MGH. **(A)** Herb-ingredient-target relationships for key targets, herbs, and ingredients of HHXYT. The integrated name of prototypes and their metabolite ID means that the metabolite is derived from the corresponding prototypes. **(B–F)** Molecular docking of HHXYT’s five key targets with corresponding compounds. The binding poses and binding sites of formononetin complexed with CYP17A1 **(B)** and HSD3B2 **(C)**; The binding poses and binding sites of glycyrrhetinic acid complexed with AR **(D)**, CYP11A1 **(E)**, and ER **(F)**.

The key targets of HHXYT for treating MGH were then obtained by combining the 4 central genes of the target network and the 3 hub genes in the first metabolite-gene module. We integrated the 7 key targets with the key herbs, prototypes (Figure 4), and their metabolites on HHXYT’s treatment to MGH, to construct the herb-ingredient-target network as shown in Figure 8A. There were 7 targets, 6 herbs, and 17 ingredients in this network. 8 of 17 ingredients were HHXYT’s prototypes absorbed in plasma, and 9 were metabolites derived from them. These herbs, ingredients, and targets constitute key substances for HHXYT to exert therapeutic effects on MGH.

At last, to verify the binding between 5 of HHXYT’s key targets and corresponding prototypes, we utilized AutoDock Vina software to conduct molecular docking. The crystal structures of 4 targets, i.e., AR (PDB ID:2q7i), CYP11A1 (PDB ID:3n9y), CYP17A1 (PDB ID:6ciz), ESR1 (PDB ID: 2qgw), which have higher resolution, were downloaded from RCSB Protein Data Bank. The predicted structure of HSD3B2 (ID: AF-P26439-F1) was downloaded from AlphaFold Protein Structure Database. The SDF files for chemical structures of the compounds (ferulic acid, formononetin, glycyrrhetinic acid, glycyrrhizic acid, isoliquiritigenin, liquiritigenin, and protocatechuic acid) were downloaded from the PubChem database. The docking scores between 14 pairs of compound-target are less than −5 kcal/mol (Table 3), suggesting that the corresponding compounds and targets have a higher binding affinity. Binding poses and binding sites for 5 pairs of compound-target interactions were shown in Figures 8B–F.

**TABLE 3:** The docking scores between HHXYT’s key targets and compounds (kcal/mol).

Target	Target structure ID	Compound	Binding score
AR	2q7i	Formononetin	−9.3
		Glycyrrhetinic acid	−17.4
		Isoliquiritigenin	−8.2
CYP11A1	3n9y	Glycyrrhetinic acid	−15.7
		Glycyrrhizic acid	−14.7
CYP17A1	6ciz	Formononetin	−8.6
		Protocatechuic acid	−5.8
ESR1	2qgw	Ferulic acid	−6.2
		Formononetin	−9.3
		Glycyrrhetinic acid	−16.6
		Isoliquiritigenin	−8.1
		Liquiritigenin	−9.5
		Protocatechuic acid	−6.1
HSD3B2	AF-P26439-F1-model_v1	Formononetin	−9.4

### 3.6 Effects of HHXYT and its Ingredient on the Testosterone Synthesis in Thecal Cells

**FIGURE 9:**
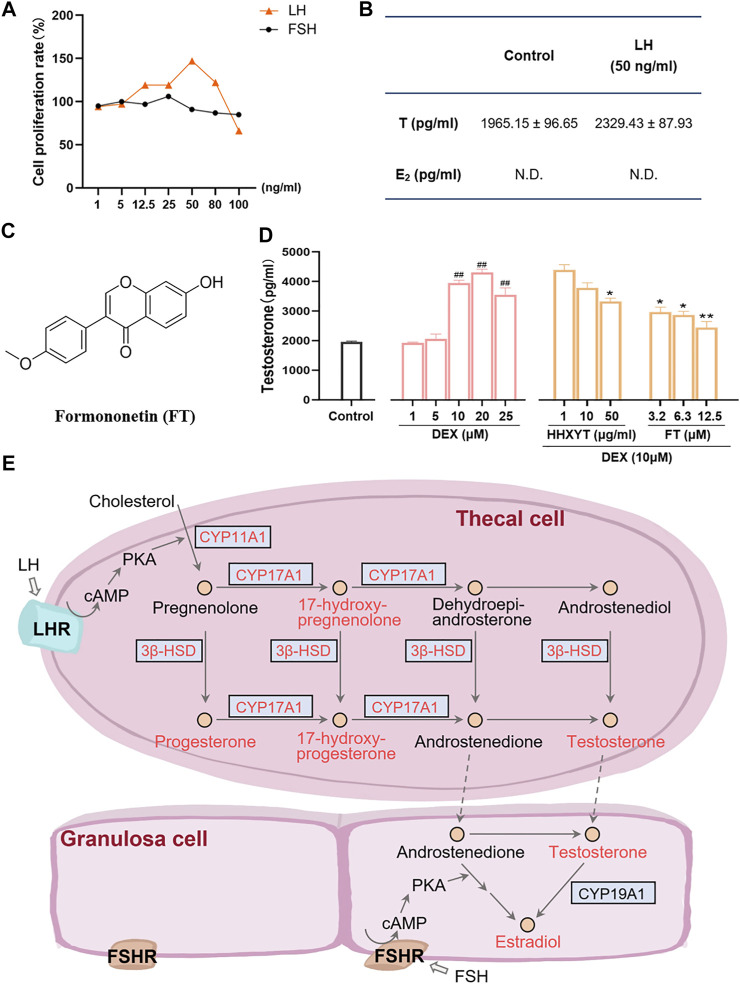
Cell-based assay to evaluate the HHXYT’s effects on testosterone synthesis in thecal cells. **(A)** The proliferation effect of luteinizing hormone (LH) and follicle-stimulating hormone (FSH) on thecal cells. **(B)** Effects of LH on the production of testosterone (T) and estradiol (E_2_) in thecal cells. **(C)** The chemical structures of formononetin (FT). **(D)** Effects of dexamethasone (DEX), HHXYT, and FT on the production of testosterone by thecal cell. *n* = 4, ##*p* < 0.01, to the Control group; *p < 0.05, **p < 0.01, compared to the DEX (10 µM) group. **(E)** Pathways for the synthesis of testosterone and estradiol by thecal cells and granulosa cells in follicles. The compounds and targets involved in this study were marked by orange-red colors.

Firstly, the cell-based assay was performed to demonstrate that the extracted and purified cells *in vitro* were thecal cells. After LH (50 ng/ml) was added, the cell proliferation rate (maximum ratio 147%) and testosterone (T) level (from 1965.15 ± 96.65 to 2,329.43 ± 87.93 pg/ml) both increased prominently (Figures 9A,B). However, after the addition of FSH, no significant changes in cell quantity were observed, and estradiol (E_2_) was not detected. It indicated that the purified cells were primarily thecal cells, with no obvious interference from granulosa cells, which were also presented in follicles of the ovary. The cell survival rate results of HHXYT and its ingredient formononetin (FT, Figure 9C) were shown in Supplementary Figure S9.

To evaluate the effect of HHXYT and its ingredient formononetin (FT) on the production of testosterone in thecal cells, thecal cells were stimulated with different concentrations of dexamethasone (DEX) and then treated with different concentrations of HHXYT and FT. The results showed that testosterone levels were markedly elevated at the DEX concentration of 10, 20, 25 µM (Figure 9D). Whereas, HHXYT (50 µg/ml) and FT (3.2, 6.3, and 12.5 µM) could distinctly inhibit the increase of testosterone level caused by DEX (10 µM) in thecal cells, suggesting that HHXYT and FT have an inhibitory effect on testosterone production in thecal cells.

## 4 Discussion

MGH is the most prevalent breast disease and the incidence of breast cancer in MGH patients is remarkably higher than that in the average person (Basree et al., 2019). However, in the current clinical treatment of MGH, hormone therapy such as tamoxifen is not suitable for long-term use with apparent ill effects and complications (Wibowo et al., 2016); operative treatment is unacceptable for patients because of its complexity. Fortunately, HHXYT has been applied in the treatment of MGH for years and its efficacy has been validated clinically (Meng et al., 2014; Pei et al., 2016).

In this study, the integrated metabolomics and network pharmacology strategy were applied to systemically reveal the mechanism of HHXYT in the treatment of MGH. Firstly, the rat model of MGH was established to reconfirm the therapeutic effect of HHXYT. HHXYT effectively inhibited the abnormal proliferation of mammary lobules, glandular bubbles, and lumens in mammary glands, according to HE staining of mammary tissue. Meanwhile, the abnormal levels of estradiol (E_2_) and progesterone (P) in serum were reversed after treatment with HHXYT. In fact, the mammary gland is closely regulated by sex hormones (Li et al., 2017). Furthermore, it has been reported that the disturbance of sex hormones, particularly estrogen and progesterone, is the primary cause and defining feature of MGH: estrogen stimulation can accelerate breast proliferation, whereas progesterone can facilitate the growth and maturation of breast acinous cells and resist the proliferation impact of estrogen (Kleinberg et al., 2011; Chang et al., 2014). In other words, high levels of E_2_ or a lack of P cause estrogen to stimulate breast epithelium for an extended period without the protection of progesterone, resulting in significant mammary tissue hyperplasia (Liu et al., 2020). As a result, the above results confirmed that HHXYT could inhibit the symptoms of MGH visibly.

To figure out active compounds which exert therapeutic effects in HHXYT, we studied the plasma of rats after oral administration of HHXYT and identified 22 prototypes and their 32 metabolites. Following that, these compounds were used for HHXYT’s putative target prediction and functional enrichment analysis by network pharmacology. 373 putative targets, 93 pathways, 6 key herbs, and 9 key prototypes of HHXYT were sought out and the most relevant of these pathways was biological processes of the endocrine, especially the synthesis of hormones (Table 2). To further explore the therapeutic mechanism of HHXYT, the analysis of serum metabolomics was performed to intuitively characterize changes in endogenous metabolites. 58 endogenous different metabolites were identified, of which 31% were steroid lipids. In addition, the most significant metabolic modules were primarily involved in steroid hormone biosynthesis, cortisol synthesis and secretion, and ovarian steroidogenesis pathways. The compounds in the second module participated in primary bile acid biosynthesis and bile secretion. Notably, all these 4 pathways were also found to be enriched by HHXYT’s putative targets. It demonstrated that metabolomic confirmed the regulation of metabolic disturbances similar to network pharmacology’s prediction results, both of which were related to hormones and bile acid biosynthesis.

Actually, bile acid was closely related to the synthesis, absorption, and metabolism of cholesterol, which was the precursor of all hormone synthesis (Capponi, 2002). As shown in Figure 9E, in the pathway of sex hormone synthesis, cholesterol was applied as a raw material to synthesize androgens, and estrogens and androgens were precursors for the production of estrogen (Williams et al., 2001). The superiority of network pharmacology is that biological networks can be constructed to assist in elucidating drug treatment mechanisms from a holistic perspective (Zhao et al., 2019; Zhao et al., 2021). Ultimately, the herb-ingredient-target network was established as the substance basis for HHXYT to exert therapeutic effects on mammary gland hyperplasia. Among the 7 key targets of HHXYT, CYP11A1, HSD3B2, and CYP17A1 were directly involved in androgen synthesis and played vital roles, meanwhile, AR and ESR1 were receptors for the direct action of androgens and estrogens, respectively. In detail, estrogen was principally generated by the synergistic effects of thecal and granulosa cells of the ovary in females. Cholesterol was converted to pregnenolone in thecal cells by CYP11A1, which was turned into androgen under the catalysis of HSD3B2 and CYP17A1, especially CYP17A1. Then androgen was generated to estrogen in granulosa cells by CYP19A1 (Imamichi et al., 2017; Burris-Hiday and Scott, 2021). According to the above results, the synthesis of sex hormones, especially androgens, was significantly regulated by HHXYT.

Therefore, it was speculated that HHXYT may reduce the level of estrogen *in vivo* by inhibiting androgen synthesis, consequently protecting the mammary gland from excessive proliferation stimulated by estrogen. There were thecal cells and granulosa cells in the follicles of the ovary. Thecal cells possessed the luteinizing hormone (LH) receptor and synthesized testosterone (T), while granulosa cells owned follicle-stimulating hormone (FSH) receptors and produced E_2_. In addition, thecal cells would promote cell proliferation and testosterone production after being stimulated by LH (Li and Hearn, 2000). To evaluate the HHXYT’s effects on the synthesis of testosterone, we extracted thecal cells from the ovaries and experimented *in vitro*. Since dexamethasone (DEX) would promote the synthesis of T in thecal cells (Ong et al., 2019), DEX was added as the model group to observe the effects of HHXYT on testosterone synthesis. The results displayed that HHXYT (50 µg/ml) could distinctly inhibit the increase of T level caused by DEX (10 µM) in thecal cells. Meanwhile, formononetin (3.2, 6.3, and 12.5 µM), a prototype of HHXYT, also exhibited the inhibiting effect on testosterone levels rising. It demonstrated that they have an inhibitory effect on testosterone production in thecal cells. In summary, these findings contribute to elucidating the treatment mechanism of HHXYT on MGH and provide data and theoretical support for the in-depth study of its mechanism.

## 5 Conclusion

In this study, through integrating metabolomics and network pharmacology analysis, our results revealed the key substances for HHXYT to exert therapeutic effects on MGH, which included 7 targets, 6 herbs, and 17 ingredients. This study illustrated that HHXYT could regulate the hormone disorder prominently and the results suggested that HHXYT might reduce estrogen-stimulated mammary gland hyperplasia by inhibiting the production of its precursor androgen. The data and theoretical support were provided for the in-depth study of HHXYT’s mechanism. In addition, this study provides new ideas for the research of the mechanism of the multi-component TCM formula.

## Data Availability

The datasets presented in this study can be found in online repositories. The names of the repository/repositories and accession number(s) can be found in the article/Supplementary Material.

## Supplementary Material

Supplementary Table S1 Original compounds identified in the rat plasma after the treatment of HHXYT.

Supplementary Table S2 Metabolites identified in the rat plasma after the treatment of HHXYT.

Supplementary Table S3 Putative targets of HHXYT’s active ingredients.

Supplementary Table S4 Diseases genes for mammary gland hyperplasia collected from GeneCards.
