# Supplementary material for: Integrated Metabolomics and Network Pharmacology Revealed Hong-Hua-Xiao-Yao Tablet’s Effect of Mediating Hormone Synthesis in the Treatment of Mammary Gland Hyperplasia
Source: Front Pharmacol. 2022 Feb 1;13:788019. doi: 10.3389/fphar.2022.788019 (PMC8846323; doi:10.3389/fphar.2022.788019)
Supplement: Supplementary file 1 [file DataSheet1.ZIP › Supplementary materials/Supplementary Table S1.docx]

| Peak | Compound ID | Compound Name | Molecular formula | Structure |
| --- | --- | --- | --- | --- |
| P1 | C1 | Desbenzoylpaeoniflorin | C16H24O10 |  |
| P2 | C2 | 1-O-β-D–Glucopyranosylpaeonisuffrone | C16H24O9 |  |
| P3 | C3 | Protocatechuic acid* | C7H6O4 |  |
| P4 | C4 | Bohenoside A | C16H30O8 |  |
| P5 | C5 | Albiflorin* | C23H28O11 |  |
| P6 | C6 | Paeoniflorin* | C23H28O11 |  |
| P7 | C7 | Ferulic acid* | C10H10O4 |  |
| P8 | C8 | Liquiritin* | C21H22O9 |  |
| P9 | C9 | Senkyunolide I* | C12H16O4 |  |
| P10 | C10 | Isoliquiritin* | C21H22O9 |  |
| P11 | C11 | Liquiritigenin* | C15H12O4 |  |
| P12 | C12 | Glyasperin B | C21H22O6 |  |
| P13 | C13 | Isoliquiritigenin* | C15H12O4 |  |
| P14 | C14 | Formononetin* | C16H12O4 |  |
| P15 | C15 | Glycyrrhizic acid* | C42H62O16 |  |
| P16 | C16 | Glycyrrhetinic acid 3-O-glucuronide* | C36H54O10 |  |
| P17 | C17 | Licoricone* | C22H22O6 |  |
| P18 | C18 | Z-ligustilide* | C12H14O2 |  |
| P19 | C19 | Gancaonin T | C24H30O5 |  |
| P20 | C20 | Licoisoflavone B* | C20H16O6 |  |
| P21 | C21 | Glycyrrhetinic acid* | C30H46O4 |  |
| P22 | C22 | Levistolide A* | C24H28O4 |  |
|  | C23 | Hydroxysafflower yellow a | C27H32O18 |  |
