# Supplementary material for: Integrated Metabolomics and Network Pharmacology Revealed Hong-Hua-Xiao-Yao Tablet’s Effect of Mediating Hormone Synthesis in the Treatment of Mammary Gland Hyperplasia
Source: Front Pharmacol. 2022 Feb 1;13:788019. doi: 10.3389/fphar.2022.788019 (PMC8846323; doi:10.3389/fphar.2022.788019)
Supplement: Supplementary file 1 [file DataSheet1.ZIP › Supplementary materials/Supplementary Table S2.docx]

| Peak | ID | Name | Formula | Structure |
| --- | --- | --- | --- | --- |
| P1 | M1 | C10H14O3 + sulfation M1 | C10H14O6S |   |
| P2 | M2 | C10H18O2 + glucuronidation M1 | C16H26O8 |      |
| P3 | M3 | Cysteine conjugate of senkyunolide I M1 | C15H21NO5S |      |
| P4 | M4 | Ferulic acid + demethylation M1 | C9H10O3 |  |
| P5 | M5 | Ferulic acid + demethylation M2 | C9H10O3 |  |
| P6 | M6 | Ferulic acid + demethylation M3 | C9H10O3 |  |
| P7 | M7 | Ferulic acid + demethylation + sulfation M1 | C9H10O6S |  |
| P8 | M8 | Ferulic acid + demethylation + sulfation M2 | C9H10O6S |   |
| P9 | M9 | Ferulic acid + demethylation + sulfation M3 | C9H10O6S |   |
| P10 | M10 | Ferulic acid + hydrogenation + sulfation M4 | C10H12O7S |  |
| P11 | M11 | Ferulic acid + hydrogenation + sulfation M5 | C10H12O7S |  |
| P12 | M12 | Ferulic acid + glucuronidation M1 | C16H18O10 |  |
| P13 | M13 | Ferulic acid + glucuronidation M2 | C16H18O10 |  |
| P14 | M14 | Ferulic acid + sulfation M1 | C10H10O7S |   |
| P15 | M15 | Formononetin + glucuronidation M1 | C22H20O10 |  |
| P16 | M16 | Formononetin + sulfation M1 | C16H12O7S |  |
| P17 | M17 | Glycyrrhetinic acid + glucuronidation M1 | C36H54O10 |  |
| P18 | M18 | Glycyrrhetinic acid + glucuronidation M2 | C36H54O10 |  |
| P19 | M19 | Glycyrrhetinic acid + oxidation M1 | C30H44O4 |  |
| P20 | M20 | Glycyrrhetinic acid + oxidation M2 | C30H46O5 |  |
| P21 | M21 | Glycyrrhetinic acid + oxidation M3 | C30H46O5 |   |
| P22 | M22 | Glycyrrhetinic acid + oxidation M4 | C30H46O5 |  |
| P23 | M23 | Glycyrrhetinic acid + oxidation M5 | C30H46O5 |   |
| P24 | M24 | Glycyrrhetinic acid + oxidation M6 | C30H44O5 |         |
| P25 | M25 | Liquiritigenin or Isoliquiritigenin + glucuronidation M1 | C21H20O10 |  |
| P26 | M26 | Liquiritigenin or Isoliquiritigenin + glucuronidation M2 | C21H20O10 |   |
| P27 | M27 | Liquiritigenin or Isoliquiritigenin + glucuronidation M3 | C21H20O10 |  |
| P28 | M28 | Liquiritigenin or Isoliquiritigenin + oxidation + sulfation M1 | C15H12O8S |  |
| P29 | M29 | Liquiritigenin or Isoliquiritigenin + sulfation M1 | C15H12O7S |   |
| P30 | M30 | Liquiritigenin or Isoliquiritigenin + sulfation M2 | C15H12O7S |  |
| P31 | M31 | Liquiritin or Isoliquiritin + glucuronidation M1 | C27H30O15 |  |
| P32 | M32 | Liquiritin or Isoliquiritin + glucuronidation M2 | C27H30O15 |  |
