# Supplementary material for: Integrated Metabolomics and Network Pharmacology Revealed Hong-Hua-Xiao-Yao Tablet’s Effect of Mediating Hormone Synthesis in the Treatment of Mammary Gland Hyperplasia
Source: Front Pharmacol. 2022 Feb 1;13:788019. doi: 10.3389/fphar.2022.788019 (PMC8846323; doi:10.3389/fphar.2022.788019)
Supplement: Supplementary file 1 [file DataSheet1.ZIP › Supplementary materials/Supplementary_Material.docx]

Supplementary Material

# Instrument parameters of HPLC-QTOF-MS/MS for metabolism analysis

LC system was considered to apply with a Shimadzu HPLC system (Nexera XR LC-20AD, Japan) and use an ACQUITY UPLC® HSS T3 (2.1 × 100 mm, 1.8 µm, Waters, USA) as the chromatographic column. The column temperature was set at 40 ℃ and the injection volume was 2 µL. The composition of the mobile phase was: phase A was mass spectrometric grade water containing 0.1% formic acid (v/v) while phase B was acetonitrile. The elution gradient at a flow rate of 0.3 mL/min was: 0-1.5 min, 5% B; 1.5-13 min, 5-100% B; 13-16.5 min, 100% B; 16.5-16.6 min, 100-5% B; 16.6-20 min, 5% B.

Mass spectrometric detection was carried out on a SCIEX Triple TOF 5600+, using the electrospray ion (ESI) source and information-dependent acquisition (IDA) mode. The TOF/MS was set at 60-1000 Da as the mass full scan range and 0.25 s as the accumulation time, while the TOF-MS/MS was set at 25-1000 Da as the mass full scan range and 0.035 s as the accumulation time. The Ion Source Gas1 (GS1) and Ion Source Gas2 (GS2) settings were 60 psi, and the Curtain Gas (CUR) was 35 psi. In positive mode: Ion Spray Voltage Floating (ISVF) was 5500 V; Temperature (TEM), 550 ℃; Collision Energy (CE), 40 V; Collision Energy Spread (CES), 10 V; Declustering potential (DP), 80 V. In negative mode: ISVF, -4500 V; TEM, 450 ℃; CE, -40 V; CES, -10 V; DP, -80 V. Furthermore, the calibration deliver system (CDS) was applied to calibrate the mass spectrometric acquisitions automatically.

# Supplementary Figures


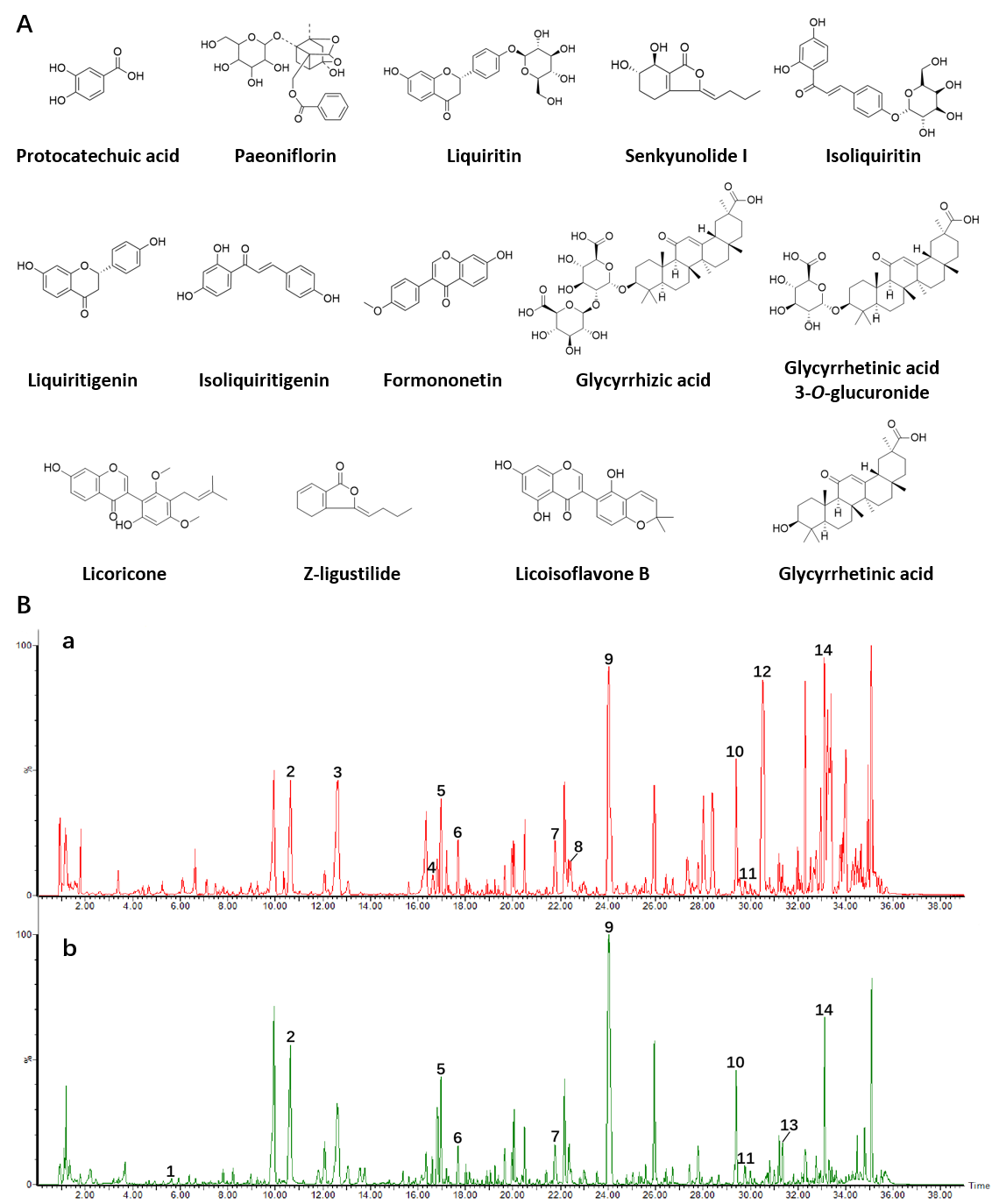


**Supplementary Figure S1.** (A) The chemical structures of 14 quality marker components of HHXYT. (B) The total ion chromatograms of HHXYT sample: (a) positive mode; (b) negative mode.

**
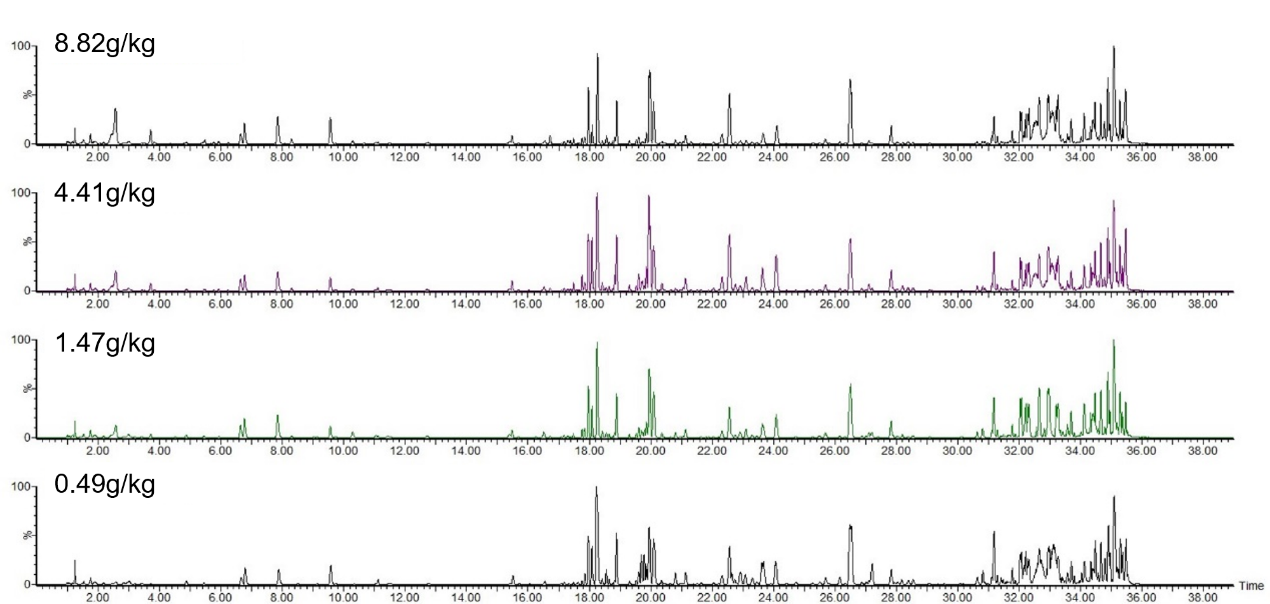
**

**Supplementary Figure S2.** Base peak ion chromatogram of plasma samples with different dosages in negative mode.

**
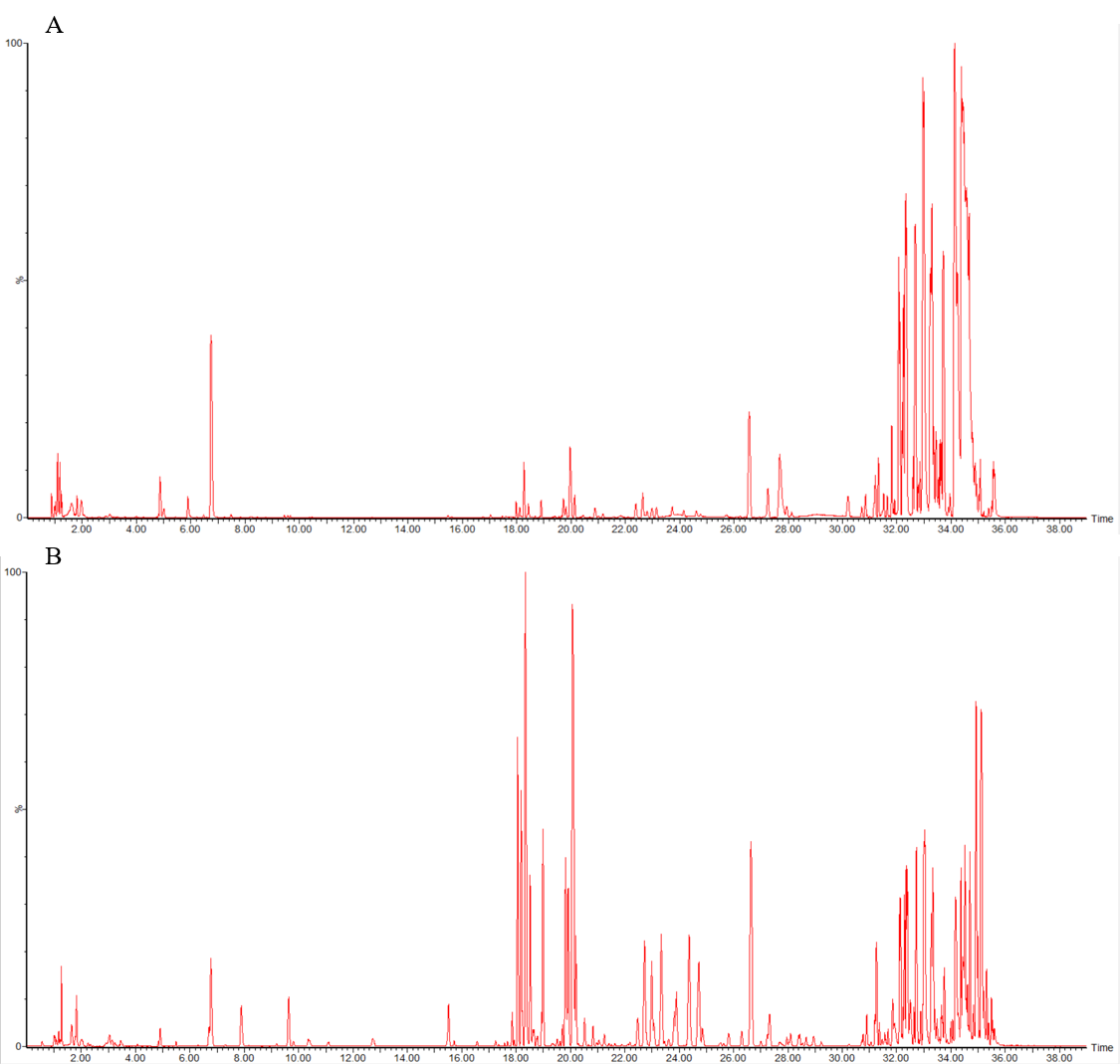
**

**Supplementary Figure S3.** Base peak ion chromatogram of blank plasma samples: (A) positive mode; (B) negative mode.


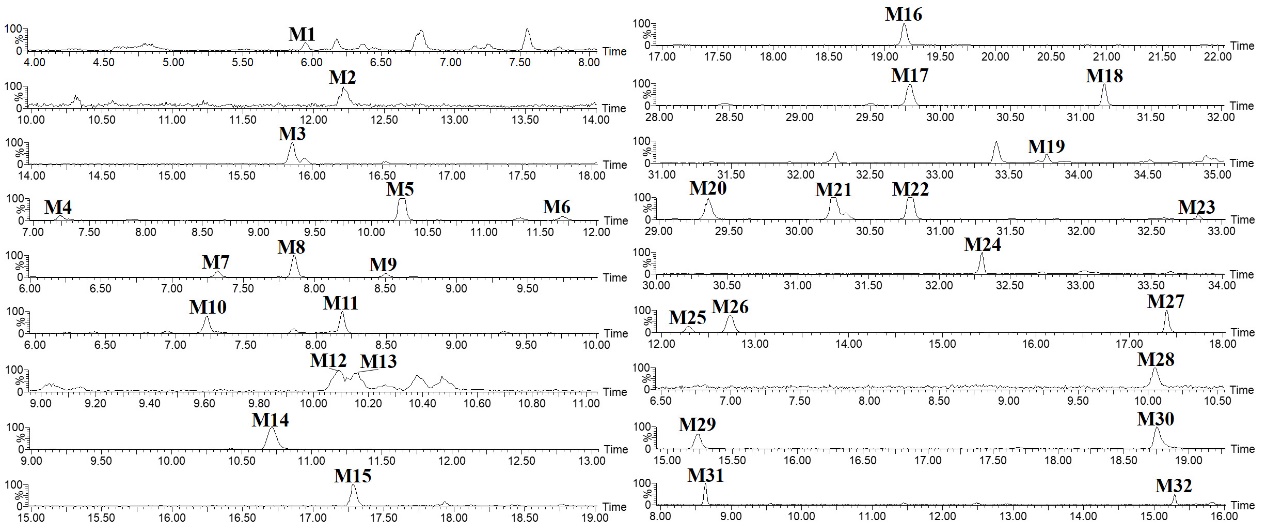


**Supplementary Figure S4.** The extract ion chromatogram of 32 metabolites after oral administration of HHXYT.


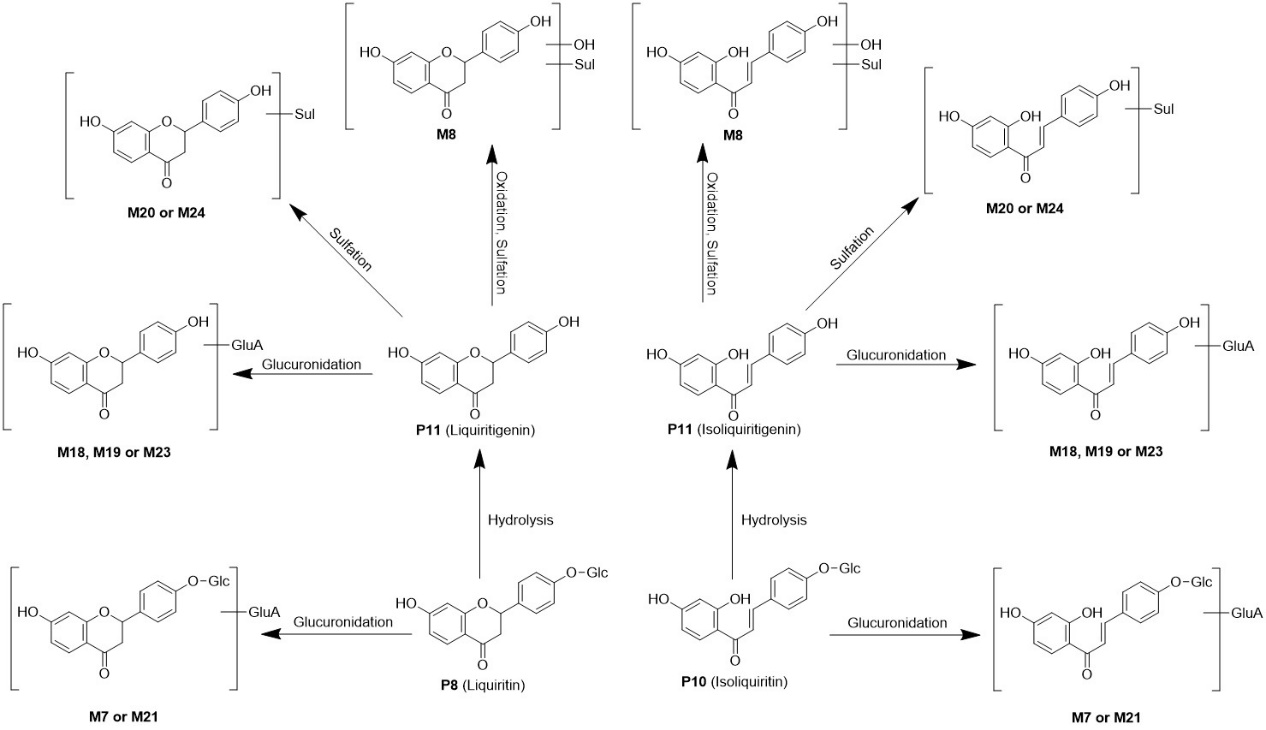


**Supplementary Figure S5.** Proposed metabolic pathways of some typical flavonoids in rat plasma after oral administration of HHXYT.


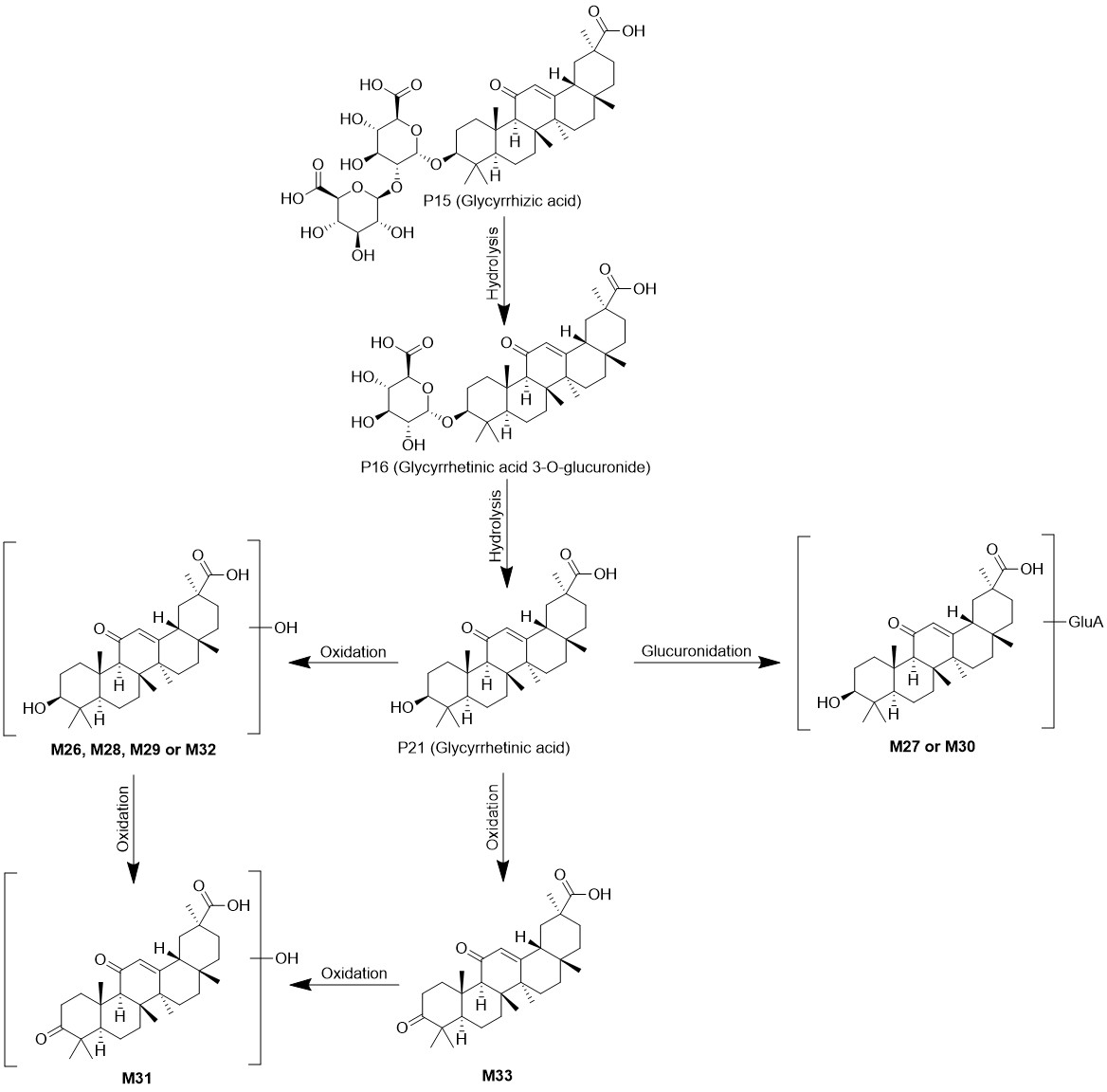


**Supplementary Figure S6.** Proposed metabolic pathways of some typical triterpene in rat plasma after oral administration of HHXYT.


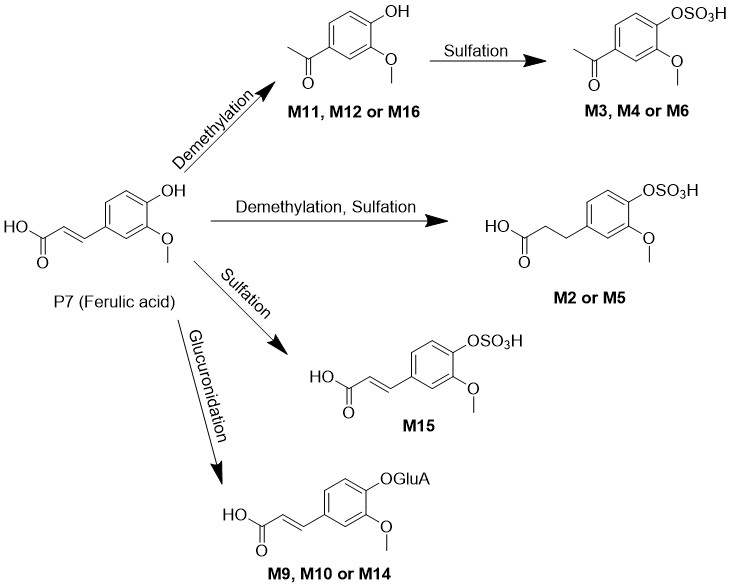


**Supplementary Figure S7.** Proposed metabolic pathways of some typical organic acid in rat plasma after oral administration of HHXYT.


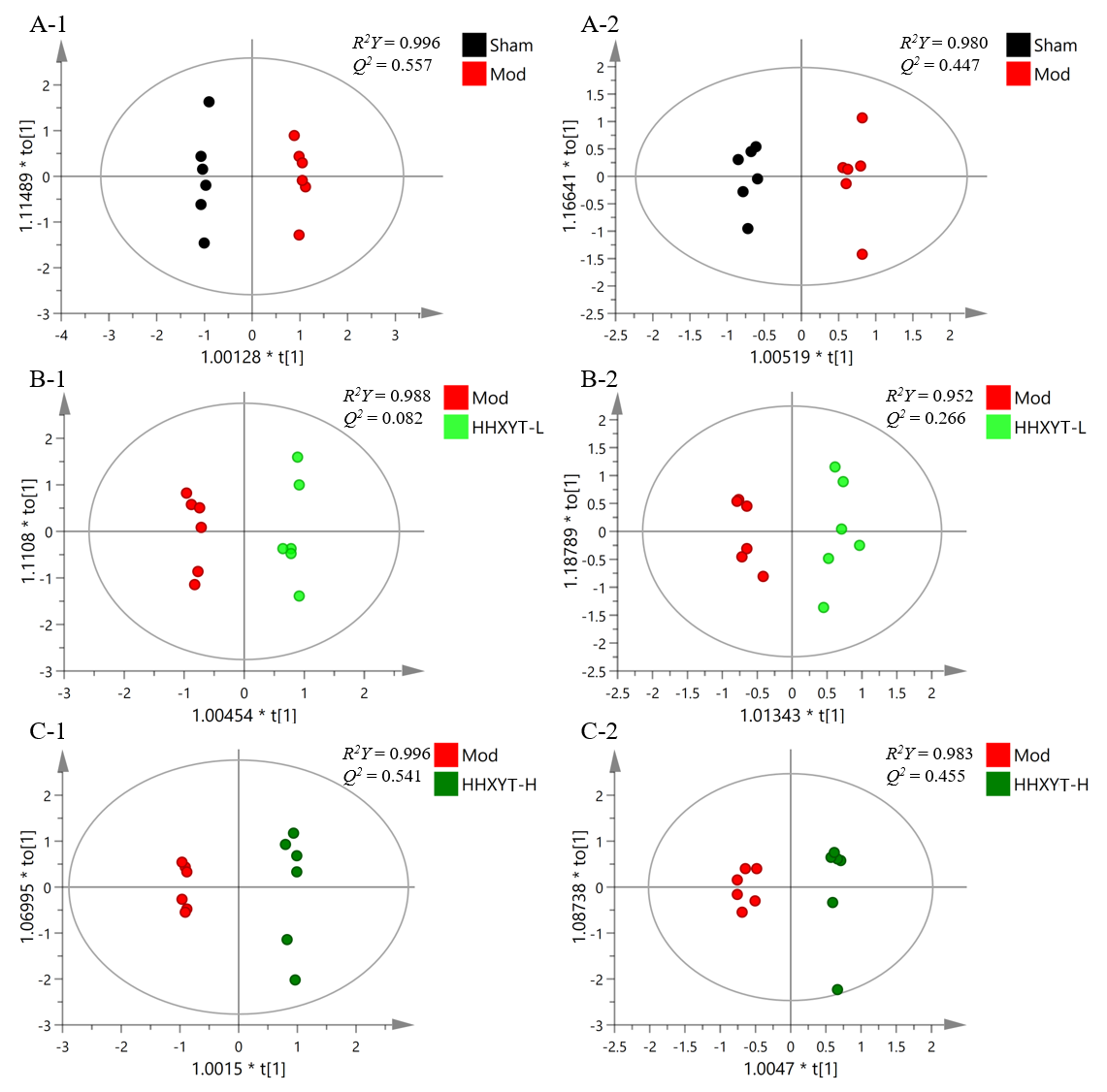


**Supplementary Figure S8.** OPLS-DA score plots of Sham/Mod (A), Mod/HHXYT-L (B), and Mod/HHXYT-H (C) group. (ESI+: A-1, B-1, and C-1; ESI-: A-2, B-2, and C-2).


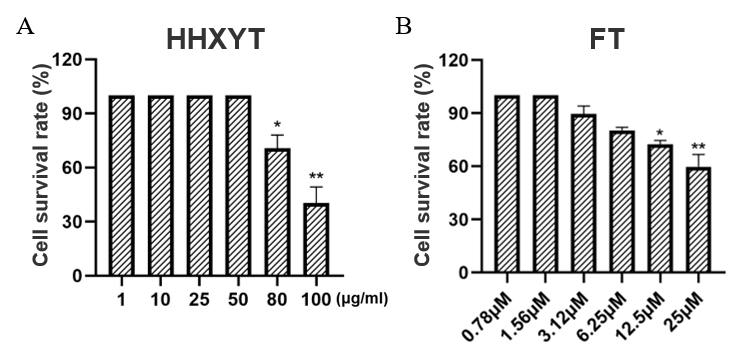


**Supplementary Figure S9.** Effects of HHXYT (A) and FT (B) on thecal cell survival.
